# Effects of Immersive Virtual Reality and Relaxation Video on Symptom Severity, Distress, and Quality of Life in Lung Cancer Patients During Initial Chemotherapy Cycles: A Three-Arm Randomised Controlled Trial

**DOI:** 10.3390/healthcare14203394

**Published:** 2026-10-12

**Authors:** Kadir Baysoy, Nur Izgu, Leyla Ozdemir, Fatma Alev Turker, Dogan Uncu, Berna Oksuzoglu

**Affiliations:** 1 Faculty of Nursing, Hacettepe University, Sıhhiye Campus, 06100 Ankara, Türkiye; akglnur@gmail.com (N.I.); leylaceyran@yahoo.com (L.O.); 2 Department of Medical Oncology, Hacettepe University Oncology Hospital, 06230 Ankara, Türkiye; aturker@hacettepe.edu.tr; 3 Department of Medical Oncology, Oncology Hospital, Ankara Bilkent City Hospital, 06800 Ankara, Türkiye; doganuncu@yahoo.com; 4 Department of Medical Oncology, Oncology Hospital, Ankara Etlik City Hospital, 06170 Ankara, Türkiye; bernaoksuzoglu@yahoo.com

**Keywords:** chemotherapy, distress, lung cancer, quality of life, randomised controlled trial, relaxation, symptom management, virtual reality

## Abstract

**Highlights:**

**What are the main findings?**

- Two 20 min sessions of immersive virtual reality or a theme-matched relaxation video during chemotherapy infusion reduced distress, tiredness, anxiety and depression and improved well-being relative to routine care, with effect sizes up to moderate.
- Differences between the two formats were small and limited to well-being, tiredness and distress. No intervention-specific effect on quality of life was found.

**What are the implications of the main findings?**

- Both immersive virtual reality and a theme-matched relaxation video are brief non-pharmacological options that may mainly relieve psychological symptoms and distress during chemotherapy.
- A theme-matched relaxation video requires only standard equipment and is an accessible option, and immersive virtual reality may offer a small additional benefit for well-being, tiredness and distress.

**Abstract:**

Background/Objectives: To examine the effects of immersive virtual reality (VR) and a theme-matched relaxation video (RV) on symptom severity, distress, and quality of life in lung cancer patients. Methods: In this open-label, three-arm, parallel-group randomised controlled trial, 315 patients were allocated (1:1:1) to the VR (*n* = 106), RV (*n* = 104), or control (CG; *n* = 105) group. During the first two chemotherapy cycles, the VR and RV groups were assigned two 20 min sessions of immersive nature-themed VR or theme-matched RV, while the CG received routine care alone. The primary outcome domain was symptom severity, assessed through within-session changes in ten individual Edmonton Symptom Assessment Scale (ESAS) symptom scores; distress and quality of life were secondary outcomes. Data were analysed with linear mixed models, and between-group differences are reported with Bonferroni-adjusted 95% confidence intervals (CI). Results: Compared with the CG, the within-session improvement was greater for eight of the ten ESAS symptoms with VR and for five with RV; the largest differences were for well-being (VR 1.26 points, 95% CI 0.98 to 1.53; RV 0.76, 0.49 to 1.03), with smaller differences for tiredness, depression, and anxiety. Distress also decreased more with VR (1.04, 0.73 to 1.34) and RV (0.73, 0.42 to 1.03) than in the CG. VR improved more than RV for well-being (0.50, 0.22 to 0.77), tiredness (0.47, 0.16 to 0.79), and distress (0.31, 0.002 to 0.62). No intervention-specific effect emerged for nausea, lack of appetite, or quality of life. Conclusions: Compared with routine care, two sessions of VR or RV improved acute symptoms, mainly well-being, tiredness, depression, and anxiety, and reduced distress, with no intervention-specific effect on quality of life. The additional benefit of VR was small and, for the ESAS symptoms, below the minimal clinically important difference, so RV may be a practical and accessible option. Study registration: ClinicalTrials.gov NCT06863623.

## 1. Introduction

Lung cancer is the most diagnosed cancer and the leading cause of cancer-related mortality worldwide, imposing a substantial clinical burden [1]. Chemotherapy is one of the primary treatment modalities for lung cancer; however, common cytotoxic agents are associated with multiple distressing symptoms such as pain, fatigue, nausea, anorexia, and neuropathy [2]. These symptoms may vary with the toxicity profiles of the agents used [3,4]. Cisplatin, for example, is highly emetogenic [5], whereas taxanes typically cause a dose-dependent sensory neuropathy [6], so different regimens produce different symptom patterns. In addition to physical symptoms, patients frequently experience anxiety and psychological distress related to treatment and disease uncertainty, which exacerbate symptom burden and deteriorate quality of life (QoL) [7].

Pharmacological options alone are often insufficient to control chemotherapy-related symptoms adequately, and holistic approaches, therefore, emphasise non-pharmacological strategies that address physiological, psychological and situational factors concurrently [8]. With advances in digital health technologies, which are increasingly used to support people with cancer [9], virtual reality (VR) has emerged as a technology-supported intervention in cancer care.

VR creates a sense of presence through computer-generated 360° environments delivered via a head-mounted display and hand controllers [10]. Its effects on symptoms are thought to arise mainly through attentional distraction and through a dampening of the physiological stress response. Attentional capacity is limited, so an absorbing virtual environment leaves less capacity for processing pain and other unpleasant bodily sensations [11,12]. This is consistent with gate control theory, which proposes that pain signals can be modulated in the spinal cord, including by descending influences from the brain [13]. In healthy volunteers exposed to thermal pain, functional magnetic resonance imaging showed reduced activity in pain-related brain regions during immersive VR [11]. In a randomised pilot trial in women receiving chemotherapy after mastectomy, salivary cortisol, the end product of the hypothalamic–pituitary–adrenal axis, was lower after the infusion in the VR group than in the control group, and blood pressure decreased with VR [14]. Recent systematic reviews and meta-analyses have reported beneficial effects of VR, including immersive VR, on anxiety, and in some reviews on depression, in patients with cancer, including those receiving chemotherapy, whereas findings for fatigue and pain have been mixed [15,16,17]. Although VR has shown beneficial effects, its routine clinical use may be limited by cost, technical requirements, staff training, implementation time, hygiene concerns, and cybersickness [12,18]. These constraints have highlighted lower-cost audiovisual interventions, delivered through standard screen-and-headphone formats, such as tablet-based guided relaxation [19], as complementary supportive care options. Such interventions may promote relaxation and redirect attention away from unpleasant symptoms, and functional imaging in healthy adults has shown that relaxation-oriented audiovisual videos activate reward-related brain regions [20]. Previous studies have also reported favourable effects of audiovisual and music-based interventions on anxiety [21,22,23] and QoL [22] among patients receiving or about to receive chemotherapy.

Despite growing evidence supporting VR and audiovisual relaxation interventions in cancer care, research directly comparing these approaches remains limited. In two paediatric trials in which the same content was presented through a head-mounted display or on a tablet, immersive delivery reduced procedural distress more [24,25], whereas another trial found no difference in anxiety at induction of anaesthesia between three-dimensional VR and a two-dimensional video [26]. Moreover, evidence specific to lung cancer patients is primarily based on pilot or quasi-experimental studies [27,28], with limited randomised controlled trials currently available [29]. To our knowledge, no randomised controlled trial has compared immersive VR and theme-matched relaxation videos (RV) across consecutive chemotherapy cycles while simultaneously examining symptom severity, distress, and QoL in lung cancer patients. Based on these gaps in the literature, this study aimed to compare the effects of immersive VR and RV interventions on symptom severity, distress, and QoL in lung cancer patients receiving chemotherapy. We hypothesised that both interventions would improve symptom outcomes compared with routine care and that immersive VR would demonstrate greater benefits than RV. Because previous findings have varied across symptom domains, each symptom of the Edmonton Symptom Assessment Scale (ESAS) was examined as a separate outcome.

## 2. Materials and Methods

### 2.1. Design, Setting, and Participants

This multicentre, three-arm, parallel-group (1:1:1), randomised controlled trial was conducted at outpatient chemotherapy units of three oncology hospitals in Ankara, Türkiye, from 15 January to 30 September 2025. Eligible participants were adults aged ≥18 years with non-small cell or small cell lung cancer at any stage and an Eastern Cooperative Oncology Group (ECOG) performance status ≤ 2 who agreed to participate and were scheduled to receive their first cycle of cisplatin/carboplatin-based chemotherapy combined with paclitaxel, docetaxel, pemetrexed, etoposide, gemcitabine, or vinorelbine [3,4].

Patients with severe cognitive or psychiatric disorders, active brain metastases, epilepsy, vertigo, chronic severe headache or dizziness, a history of motion sickness, severe visual or hearing impairments, or intervention-related phobias were excluded. The intervention protocol was refined in pilot testing with 10 lung cancer patients, whose feedback helped determine the 20 min session duration, the two-cycle schedule, and the selection of the “Walking in the Forest” (Green Meadows) and “Sitting on the Beach” (Blue Ocean) scenarios from six candidate environments for both VR and RV. Participants involved in the pilot phase were not included in the main study sample.

### 2.2. Sample Size Estimation

Sample size was estimated using an a priori power analysis with G*Power 3.1.9.7 (Heinrich-Heine-Universität Düsseldorf, Düsseldorf, Germany) [30]. The calculation used the G*Power F-test procedure for repeated-measures analysis of variance (ANOVA; within-between interaction) with three groups and two measurements. Drawing on the findings of Li et al. [31] and on systematic reviews of immersive VR during chemotherapy [15,16], a small-to-medium group × time effect was conservatively assumed for the ESAS symptom scores. Accordingly, based on a small-to-medium effect size (Cohen’s *f* = 0.15; correlation among repeated measures *r* = 0.50; nonsphericity correction ε = 1) with α = 0.05 and 1 − β = 0.95, a minimum total sample size of 177 participants (59 per group) was required. To account for an anticipated attrition rate of 35%, the midpoint between the 26% primary-endpoint and 44% end-of-study attrition rates reported in supportive and palliative oncology trials [32], the target sample size was increased to 273 participants (91 per group), and recruitment continued throughout the enrolment period, yielding 315 randomised participants.

### 2.3. Randomisation, Allocation, and Blinding

Participants were stratified according to cancer stage (Stage I–II/early/limited vs. Stage III–IV/advanced/extensive) and chemotherapy protocol to promote baseline symptom balance [33] and randomised to the VR, RV, or control groups in a 1:1:1 ratio using restricted randomisation with a maximum tolerated imbalance of five rather than permuted blocks; randomisation was not stratified by centre. The allocation sequence was computer-generated in R and held by a researcher who was not involved in intervention delivery or data collection, and each allocation was obtained from that researcher by telephone at enrolment. Due to the nature of the interventions, participant, implementer, and outcome assessor blinding were not feasible, and the trial was therefore open label. During the analysis phase, the intervention groups were replaced with unpredictable, non-relational numerical codes to reduce potential interpretation bias.

### 2.4. Interventions

All sessions were delivered by a single trained researcher across the three centres to ensure procedural consistency. The researcher, a doctoral candidate and research assistant in nursing with ten years of professional experience, had used the device extensively while selecting and testing the intervention content, and completed the device training and rehearsed the full session protocol, including the safety checks, before the first patient was enrolled. Session duration, timing within the infusion and delivery conditions were the same at all three centres. VR content was selected from the nature-based virtual environments of the Nature Treks VR application (Greener Games, UK), with nature sounds and background music, in which participants could look around freely and move using the hand controllers. RV content was developed in a slide-video format using royalty-free Pixabay images with relaxing background music and ambient sounds obtained from YouTube and was matched to the VR environments in theme, sequence and duration rather than in visual form; the videos contained no narration or breathing instruction. All intervention materials were reviewed by an expert panel and assessed for psychiatric appropriateness before implementation. The experts evaluated the appropriateness of both interventions for relaxation and distraction, as well as the thematic matching of the RVs, yielding an overall content validity index (S-CVI/Ave) ≥ 0.90 (Supplementary Material, Table SA.2).

**Figure 1:**
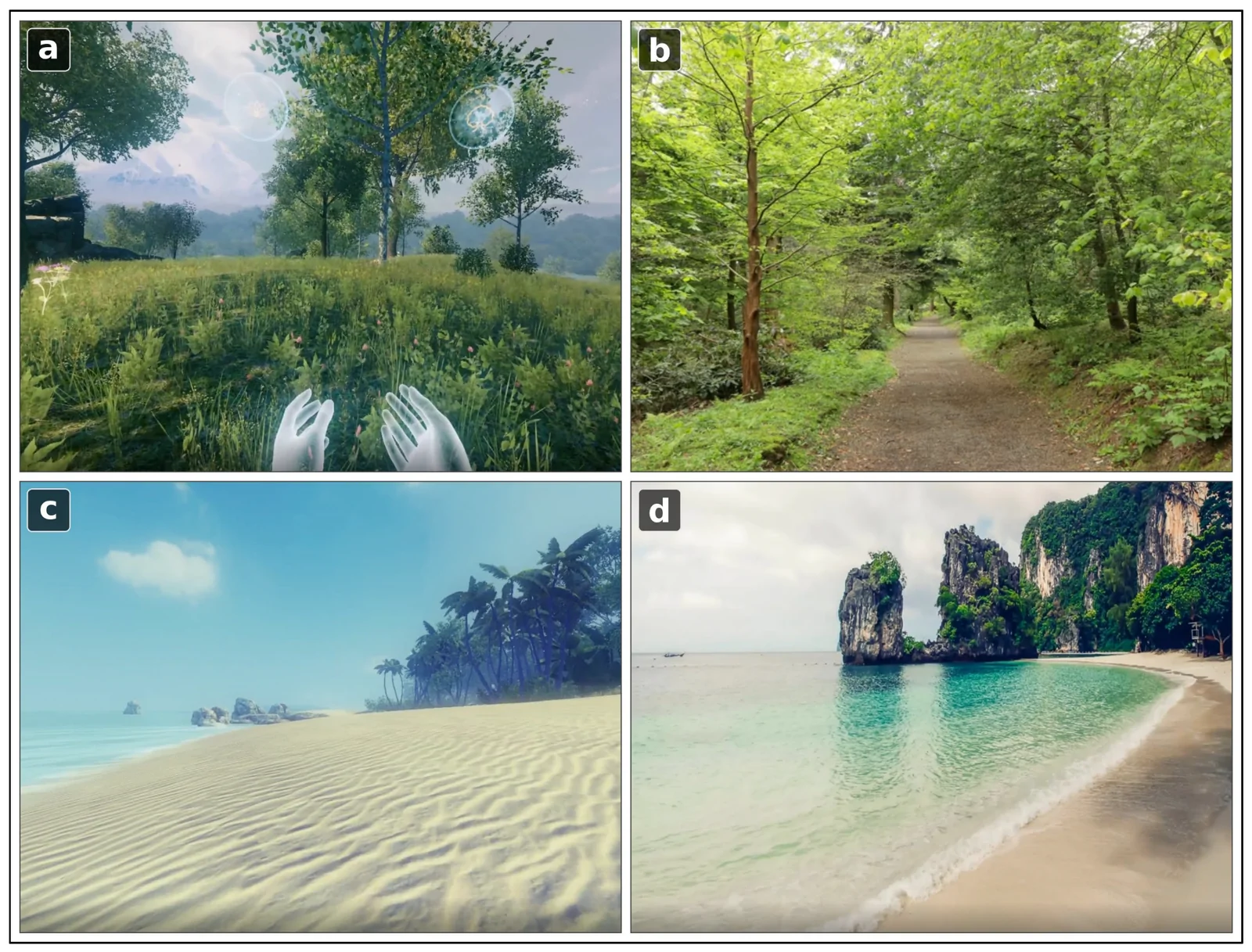
Content samples from the study interventions (Forest and Beach themes). (**a**,**c**) First-person views from the immersive virtual reality (VR) condition (screenshots from Nature Treks VR, Greener Games); (**b**,**d**) screenshots from the theme-matched relaxation videos (RV) presented on standard screens (images from Pixabay).

Both interventions lasted 20 min per session and were administered, in addition to routine care, once during each of the first two chemotherapy cycles, at least 30 min after infusion initiation and, generally, during the middle phase of the infusion, so that acute infusion-related reactions could be monitored beforehand and the session fell clear of the start and the end of treatment. The VR group received the intervention using a Meta Quest 2 head-mounted display (Meta Platforms, Inc., Menlo Park, CA, USA) and hand controllers, running the commercially available version of the application purchased from the Meta Quest Store, which provided an immersive interactive experience. The RV group viewed theme-matched videos on screens ≥ 12.4 inches using Bluetooth headphones in a non-immersive audiovisual format (Figure 1). At the start of each session, the researcher adjusted the audio level and the participant’s position for comfort and did not intervene thereafter; participants in both groups were told beforehand that they could stop at any point. The head-mounted display, controllers, and headphones were disinfected with 70% alcohol before and after each use, and single-use pads were applied to the headphones. The control group (CG) received routine care alone, including monitoring of vital signs and chemotherapy-related side effects, with no additional distraction or supportive intervention. The procedure in each group is set out step by step in Supplementary Material, Table SA.3.

### 2.5. Outcome Measures and Data Collection

The primary outcome domain was symptom severity, assessed as the within-session change (post-session minus pre-session score) in each of the ten ESAS symptoms, so that negative values indicate improvement. The primary hypothesis was that this change would differ between the study arms; it was tested separately for each symptom, and the ten tests were treated as one family for multiplicity control (Section 2.7).

The ESAS was developed as a simple method for assessing common symptoms in palliative care patients [34]. The Turkish version used in this study comprises twelve items: pain, tiredness, nausea, depression, anxiety, drowsiness, lack of appetite, shortness of breath, numbness, well-being, skin changes and mouth ulcer [35]. All items are rated from 0 to 10, with lower scores indicating a better state; this also applies to well-being, for which 0 denotes the best and 10 the worst possible well-being.

All twelve items were administered at every assessment. Two items, skin changes and mouth ulcer, which record mucocutaneous changes rather than momentary symptoms, did not change within any session. When they were entered into the within-session model, no estimate could be obtained because their change was zero in every session; they are therefore reported descriptively in Supplementary Table SB.2. Inferential analyses of within-session change were therefore conducted for the remaining ten symptoms: pain, tiredness, nausea, depression, anxiety, drowsiness, lack of appetite, shortness of breath, numbness, and well-being. Cronbach’s α for the ten symptoms was 0.69, 0.70, 0.71 and 0.73 at the four assessments, and 0.68, 0.69, 0.70 and 0.72 when these two items were also included (Supplementary Table SB.2).

Secondary outcomes included changes in distress and QoL, measured using the National Comprehensive Cancer Network (NCCN) Distress Thermometer (NCCN-DT) and the European Organization for Research and Treatment of Cancer Quality of Life Questionnaire-Core 30 (EORTC QLQ-C30), respectively. The NCCN-DT is a validated screening tool for distress scored from 0 to 10, with higher scores indicating greater distress and scores ≥ 4 indicating clinically relevant distress [36]. The EORTC QLQ-C30 is a cancer-specific quality-of-life instrument [37] that has been validated in the Turkish population [38]. This 30-item multidimensional tool comprises five functional scales (physical, role, cognitive, emotional, and social), three symptom scales (fatigue, pain, and nausea/vomiting), six single items (dyspnoea, insomnia, appetite loss, constipation, diarrhoea, and financial difficulties), and a global health status/QoL scale. In the analyses, the global health status/QoL subscale served as the primary quality-of-life indicator, while the EORTC QLQ-C30 summary score was used as a complementary measure. The summary score was calculated as the mean of the subscales except for the global health status/QoL and financial difficulties, with symptom scores reversed so that higher scores represent better QoL. Cronbach’s α coefficients for the global health status/QoL and summary score in this sample were 0.86 and 0.85 at baseline, and 0.83 and 0.85 at the end of the study, respectively.

Sociodemographic and clinical characteristics were collected using the 18-item Patient Information Form, whereas adverse events and patient experience data were recorded using the Patient Follow-up Form.

The ESAS and NCCN-DT (current-state ratings) were administered at four time points: before (T1 and T3) and after (T2 and T4) each intervention session across two chemotherapy cycles. Because the EORTC QLQ-C30 refers to the preceding week, it was not repeated around each session and was administered only before the first intervention (T1) and after the final intervention (T4). The same measurements were applied at corresponding time points in the CG (Supplementary Material, Table SA.1).

### 2.6. Ethical Considerations

The trial is reported in accordance with the CONSORT 2025 [39] and TIDieR [40] guidelines. The study protocol was approved by the Hacettepe University Ethics Committee of Clinical Trials (Decision No: 2022/17-01 (KA-22070), Date: 10 October 2022) and the Turkish Medicines and Medical Devices Agency (TİTCK). Institutional permissions were obtained from the study settings before study initiation, and the trial was prospectively registered at ClinicalTrials.gov (NCT06863623). The study was conducted in compliance with the Declaration of Helsinki. All participants provided written informed consent before enrolment. Participants were observed throughout each session and asked at its end whether they had experienced any problem, such as cybersickness symptoms, general discomfort or an infusion-related reaction, and any reported problem was recorded. No personally identifiable information beyond contact details was collected, and data were processed anonymously. Participants were also informed that they could withdraw at any time without consequence.

### 2.7. Statistical Analysis

Analyses were performed in IBM SPSS Statistics 31.0 (IBM Corp., Armonk, NY, USA) with R 4.6.1 (R Foundation for Statistical Computing, Vienna, Austria) integration [41]. All randomised participants were analysed in their assigned groups using all available assessments, consistent with the intention-to-treat principle. Continuous variables were summarised as mean ± SD or median (25th–75th percentile), and categorical variables as frequencies and percentages. Baseline differences between groups were tested with one-way ANOVA and chi-square tests, reported for completeness and not used to select covariates.

Linear mixed models were used as the primary analytical approach because they use all available assessments without imputation, provide valid estimates when data are missing at random and model the correlation between repeated assessments of the same participant directly [42,43]. The study used a 3 (group) × 2 (chemotherapy cycle) × 2 (pre- versus post-session assessment) design, comprising 315 first-cycle and 210 second-cycle sessions. Group, cycle and assessment point and all their interactions were entered as fixed effects, and the four assessments of each participant were modelled with an unstructured covariance matrix. Models were fitted by restricted maximum likelihood with Satterthwaite degrees of freedom. Model adequacy was checked through convergence, the positive definiteness of the estimated covariance matrices and residual diagnostics.

The pre-session assessment of each cycle, T1 in the first and T3 in the second, served as the baseline for the within-session change in that cycle. The group × time interaction evaluated whether the within-session change differed between groups, averaged over the two cycles, and the group × cycle × time interaction examined whether this difference was consistent across cycles. Pairwise comparisons between groups were estimated from the same model. For the EORTC QLQ-C30, a group × time model with the baseline and end-of-study assessments was used.

Pairwise comparisons were Bonferroni-adjusted for the three comparisons within each outcome, and their 95% confidence intervals (CIs) were adjusted in the same way. Group × time interaction tests for the ten ESAS symptoms used Benjamini–Hochberg false-discovery-rate (FDR) correction; distress, the global health status/QoL score and the EORTC QLQ-C30 summary score were each tested as a single outcome, so no multiplicity correction was required. Effect sizes, standardised by the pooled within-group standard deviation at baseline, were Cohen’s *d* for pairwise comparisons, calculated from the between-group difference in within-session change [44], and Cohen’s *f* for the interaction effects, calculated from the root-mean-square deviation of the three arms’ model-estimated changes around their mean [45]. A one-point change was used as the minimal clinically important difference for a single ESAS symptom [46].

A complete-case analysis of the 210 participants with all four assessments and separate models for each chemotherapy cycle were added as sensitivity analyses (Supplementary Material, Table SC.1,2).

## 3. Results

### 3.1. Participant Flow and Baseline Characteristics

**Figure 2:**
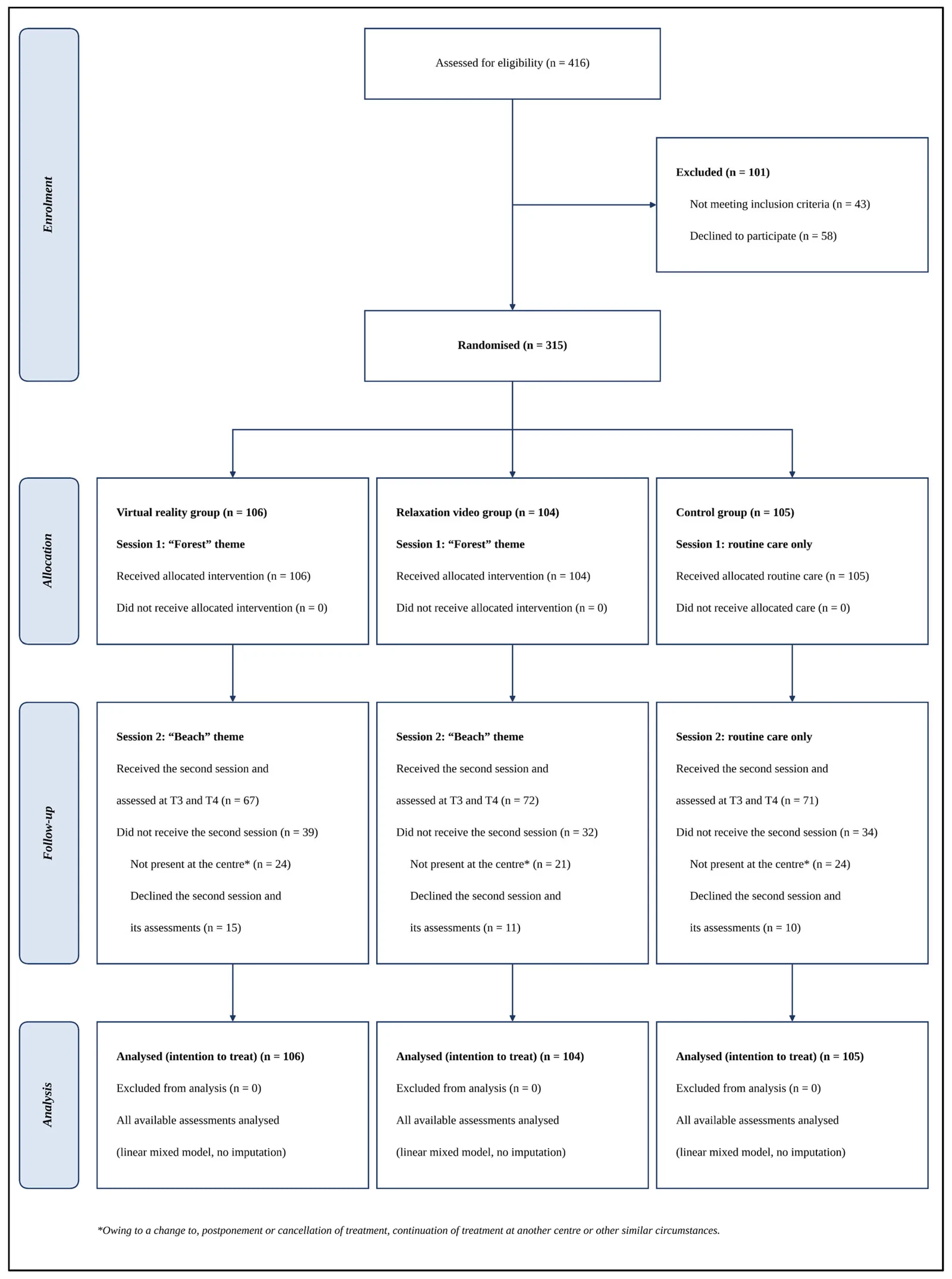
CONSORT flow diagram.

During the recruitment period, a total of 416 individuals were screened for eligibility. Of these, 101 patients were excluded due to not meeting the inclusion criteria (*n* = 43) and declining to participate (*n* = 58). Of 315 patients randomised (VR *n* = 106; RV *n* = 104; CG *n* = 105), all completed the first intervention session with complete pre- and post-session data. A second session was completed by 210 participants (66.7%; VR *n* = 67, 63.2%; RV *n* = 72, 69.2%; CG *n* = 71, 67.6%), and 105 (33.3%) did not receive it (VR *n* = 39; RV *n* = 32; CG *n* = 34). The second session was not received either because the participant was not present at the centre at the time of the session, owing to a change to, postponement or cancellation of treatment, continuation of treatment at another centre or other similar circumstances (VR *n* = 24; RV *n* = 21; CG *n* = 24), or because the participant declined the second session and its assessments (VR *n* = 15; RV *n* = 11; CG *n* = 10). All randomised participants were retained in the intention-to-treat analysis (Figure 2). The sample was predominantly male (78.7%), with 54.3% aged ≥ 65 years. The most common platinum-based chemotherapy regimens included taxane (28.9%), etoposide (23.8%), and weekly and radiotherapy-concurrent taxane (23.5%). Baseline sociodemographic, clinical, and outcome characteristics were comparable across groups (Table 1; Supplementary Material, Table SB.1). The proportion assessed at the second session did not differ across groups (χ^2^ = 0.92; *p* = 0.631), and participants who did and did not receive the second session did not differ on the baseline measures or, in the sample as a whole, on the within-session change in the first session (Supplementary Material, Table SB.3).

### 3.2. Effects of Interventions

Unadjusted descriptive statistics (mean ± SD) and the corresponding per-timepoint within- and between-group comparisons across the four assessment points (T1–T4) are provided in Supplementary Material (Table SB.1). All confirmatory time-dependent inferences below are based on the linear mixed models.

#### 3.2.1. Primary Outcome Domain: ESAS Symptoms

In the linear mixed models combining both intervention sessions, the group × time interaction was significant after FDR correction for eight of the ten symptoms. The interaction effect was largest for well-being (*f* = 0.24), followed by tiredness, depression and anxiety (*f* = 0.12 each), and smaller for pain (*f* = 0.07), numbness (*f* = 0.05), drowsiness (*f* = 0.04) and shortness of breath (*f* = 0.03). Nausea and lack of appetite did not show a significant group × time interaction. The group × cycle × time interaction was non-significant for all outcomes (Table 2); estimates averaged over the two cycles are therefore reported, with cycle-specific estimates in Supplementary Table SC.2.

Within-session changes and the between-group mean differences (MD) in those changes are shown in Table 3. Compared with the CG, the within-session improvement was greater with VR for pain, tiredness, depression, anxiety, drowsiness, well-being, shortness of breath and numbness, and with RV for tiredness, depression, anxiety, drowsiness and well-being. The largest differences were for well-being (VR vs. CG: MD 1.26, 95% CI 0.98 to 1.53, *d* = 0.59; RV vs. CG: MD 0.76, 0.49 to 1.03, *d* = 0.36), with further differences for tiredness (VR vs. CG: MD 0.87, 0.55 to 1.19, *d* = 0.29; RV vs. CG: MD 0.40, 0.08 to 0.71, *d* = 0.13), depression (VR vs. CG: MD 0.74, 0.45 to 1.04; RV vs. CG: MD 0.58, 0.28 to 0.87) and anxiety (VR vs. CG: MD 0.74, 0.45 to 1.04; RV vs. CG: MD 0.56, 0.26 to 0.86). The differences in the remaining physical symptoms were smaller: pain (VR vs. CG: MD 0.35, 0.10 to 0.60), drowsiness (VR vs. CG: MD 0.27, 0.10 to 0.44; RV vs. CG: MD 0.17, 0.002 to 0.34), numbness (VR vs. CG: MD 0.16, 0.02 to 0.30) and shortness of breath (VR vs. CG: MD 0.14, 0.01 to 0.27). Neither intervention differed from the CG for nausea or lack of appetite. The VR versus RV comparison reached significance after Bonferroni adjustment for well-being (MD 0.50, 95% CI 0.22 to 0.77) and tiredness (MD 0.47, 0.16 to 0.79). Trajectories across the four assessment points and the corresponding effect sizes are shown in Supplementary Material, Figure SD.1,2.

#### 3.2.2. Secondary Outcomes: Distress Level and Quality of Life

A significant group × time interaction was observed for distress (*F*(2, 302.6) = 34.46, *p* < 0.001; Table 2). The group × cycle × time interaction was not statistically significant. Within-session reductions were 1.08, 0.77, and 0.04 points in the VR, RV and CG arms, respectively. Both VR and RV showed significant effects compared with the CG (VR vs. CG: MD 1.04, 95% CI 0.73 to 1.34, *d* = 0.41; RV vs. CG: MD 0.73, 95% CI 0.42 to 1.03, *d* = 0.29; both *p* < 0.001) (Table 3; Supplementary Material, Figure SD.1,2). The difference between VR and RV was also significant (MD 0.31, 95% CI 0.002 to 0.62, Bonferroni-adjusted *p* = 0.048, *d* = 0.12).

No intervention-specific effect on quality of life was demonstrated (Table 4). The global health status/QoL score increased in all three groups (VR +7.58, RV +5.15 and CG +4.77 points; time effect *F*(1, 251.5) = 15.05, *p* < 0.001), but the increase did not differ between groups (group × time *F*(2, 251.4) = 0.34, *p* = 0.715). The summary score did not change over time and did not differ between groups (group × time *F*(2, 218.7) = 1.45, *p* = 0.236). The between-arm differences in change were 2.80 points for VR versus CG (95% CI −6.11 to 11.72) and 0.38 for RV versus CG (−8.44 to 9.19) on the global health status/QoL score, and −3.38 (−8.28 to 1.51) and −1.03 (−5.84 to 3.79) on the summary score (Bonferroni-adjusted; positive values favour the first-named arm).

In the complete-case analysis of the 210 participants with all four assessments, the between-group differences were close to those of the primary analysis, and every comparison that was significant in the primary analysis remained significant except the VR versus CG differences in shortness of breath and numbness, for which the group × time interaction also no longer reached significance after FDR correction (Supplementary Table SC.1). Estimates for each chemotherapy cycle separately, including the first cycle, which contains no missing data, are given in Supplementary Table SC.2; every comparison that was significant in the primary analysis pointed in the same direction in both cycles.

### 3.3. Adverse Events

In the VR group, 1 of 106 participants (0.9%) reported mild headache and 1 (0.9%) mild dizziness; in the RV group, 1 of 104 (1.0%) reported mild sadness. No adverse events were reported in the CG. None of these events required discontinuation of the intervention, and no serious adverse event was observed. Because adverse events were elicited by a single open question at the end of each session rather than with a validated instrument such as the Simulator Sickness Questionnaire [47], mild and transient cybersickness may have been under-recorded.

## 4. Discussion

Individuals with lung cancer receiving chemotherapy experience a substantial symptom burden, yet adequately powered randomised evidence on digital non-pharmacological interventions in this population has been limited. This three-arm randomised controlled trial of 315 patients in three oncology centres addresses this gap by showing that both immersive VR and a theme-matched RV were associated with greater improvements than routine care in distress and in several symptoms (eight of the ten with VR and five with RV). Participants in both intervention groups also received additional attention from the researcher, which may have contributed to the difference from routine care and should be considered when interpreting it.

Regarding the primary outcomes, both VR and RV reduced tiredness, depression, and anxiety and improved well-being compared with routine care. These findings are consistent with previous research reporting beneficial effects of immersive VR on anxiety and other psychological symptoms in patients with cancer [15,16] and of nature videos on emotional state in oncology waiting rooms [48]. A possible explanation is that audiovisual distraction and relaxation may help redirect attention away from distressing treatment-related experiences. In a randomised cross-over study, exposure to a natural environment reduced distress and increased mindfulness compared with an urban setting [49]. A one-point change has been proposed as the minimal clinically important difference for a single ESAS symptom [46]. Within the groups, this threshold was reached only for well-being in the VR group (VR −1.31; RV −0.82; Table 3), and among the between-group differences only well-being was of comparable magnitude (MD 1.26).

In contrast to the effects on well-being, tiredness and the affective symptoms, VR and RV showed smaller benefits for the other physical symptoms. Differences against routine care reached significance for pain, drowsiness, numbness and shortness of breath with VR and for drowsiness with RV, but amounted to 0.4 points or less, below the one-point threshold for a single symptom, and those for shortness of breath and numbness did not remain significant in the complete-case analysis. Nausea and lack of appetite did not respond; they may be driven more strongly by treatment-related physiological mechanisms, and nausea in particular was rated close to the lower end of the scale at the start of the sessions, which leaves little room for a within-session change to appear. This is consistent with evidence suggesting that immersive VR interventions during chemotherapy may have more consistent effects on anxiety than on pain [15]. Future studies may be needed to determine whether longer or repeated exposure to these interventions can produce clinically meaningful improvements in the physical symptoms.

Turning to the secondary outcome of distress, significant reductions were observed in both intervention groups compared with the CG, with the larger reduction in the VR arm. The difference between VR and RV was very small (MD 0.31, *d* = 0.12) and is consistent with Bachelard et al. [50], in which the same nature content delivered via a head-mounted display rather than a tablet produced greater reductions in distress and stronger feelings of presence, although that study was not randomised and included 42 patients. The reductions relative to routine care are in line with Li et al. [31], who reported lower distress at the end of a 3-month intervention and at 6-month follow-up among patients with breast cancer receiving VR-based interventions. While Li et al. [31] evaluated longer-term outcomes, the present study suggests that beneficial effects on distress may also be detectable within minutes of intervention delivery. Similar findings have been reported by Fabi et al. [51], who observed within-group reductions in state anxiety among patients with breast and ovarian cancer receiving VR during chemotherapy. An umbrella review by Hao et al. [17] similarly reported significant pooled effects of VR on anxiety and depression across oncology populations. To our knowledge, no minimal clinically important difference has been established for the NCCN-DT.

Regarding the QoL findings, a cautious interpretation is warranted. The global health status/QoL score increased in all three groups, whereas the summary score did not change. The group × time interaction was not significant for either score, so the improvement cannot be attributed to the interventions. The absence of an intervention-specific effect is consistent with Li et al. [31], in whose trial VR did not significantly improve global health status/QoL relative to the control group at 3 or 6 months, although several functional scales improved, and with the systematic review by Alvarado-Omenat et al. [52], in which only two of the six included studies assessed QoL and results varied with the sample and the intervention protocol. Longer follow-up periods and studies specifically powered to evaluate quality of life as a primary outcome, with the instrument administered on a schedule that matches its recall window, may help clarify whether intervention-related benefits emerge over time.

Comparing the two formats, the difference reached significance for well-being (MD 0.50, *d* = 0.23), tiredness (MD 0.47, *d* = 0.16) and distress (MD 0.31, *d* = 0.12). Previous comparisons of immersive and non-immersive delivery, mostly during paediatric procedures, have given mixed results. Ryu et al. [24] reported less distress with immersive delivery than with a matched tablet video during chest radiography, and Lee et al. [25] reported less pain and distress during venipuncture, whereas Samnakay et al. [26] found no difference in anxiety at induction of anaesthesia. In our trial, researcher contact, session length and content theme were the same in both intervention arms, so attention alone is unlikely to explain the difference between them. The formats did, however, differ in visual form and immersion, and novelty, expectancy and participant preference may also have played a part. Overall, the additional benefit of immersive delivery was modest. It was limited to well-being, tiredness and distress and, for the two ESAS symptoms, remained below the minimal clinically important difference proposed for a single symptom. The absence of a larger difference is not evidence of equivalence, since the trial was not designed as an equivalence or non-inferiority study. Taken together, these findings suggest that RV delivered on ordinary equipment is a pragmatic and accessible option for most of the symptoms studied, and that a head-mounted display may be considered where well-being, tiredness or distress is prioritised or where the patient prefers the immersive format.

### 4.1. Limitations

Several limitations should be acknowledged. Blinding of participants, the implementer and outcome assessors was not feasible, although the analyses were conducted under masked group codes. Because participants in both intervention groups knew that they were receiving an additional intervention, expectancy and novelty effects may have contributed to the comparison with routine care, and the matched video controlled for attention and theme but not for the expectation attached to a head-mounted display. A single researcher delivered all sessions, which ensured procedural consistency but does not allow the effect of the technology to be separated from that of the person delivering it, and fidelity was not scored independently. Follow-up covered only two consecutive chemotherapy cycles, so longer-term outcomes were not assessed. One-third of participants did not receive the second session. Non-receipt was similar across arms, but it reduces precision for the second cycle, could bias the estimates if the missing-at-random assumption does not hold and may partly reflect the burden of research participation during active treatment. The trial was powered for a small-to-medium effect, so small differences in individual symptoms may have been missed and non-significant comparisons should not be read as evidence of no effect. The quality-of-life questionnaire refers to the preceding week but was administered immediately after a session, so it was not well suited to detecting an immediate effect. Adverse events were recorded by direct questioning rather than with a validated cybersickness instrument, so mild transient effects may have been under-recorded. Participants were recruited from three oncology centres in one city; most were male, more than half were aged 65 years or older, and all received platinum-based chemotherapy, which limits generalisation to younger patients, women, patients with poorer performance status, other regimens and non-specialist or resource-limited settings. The regimens also differed in toxicity profile, and because the regimen, histological and stage subgroups would have been small once divided across the three arms, the findings apply to the regimens as a whole rather than to any single regimen. Finally, no cost data were collected, and the intervention content was standardised rather than adapted to individual preferences.

### 4.2. Implications for Clinical Practice and Future Research Directions

Both immersive VR and theme-matched RV reduced distress and improved well-being, tiredness, depression and anxiety compared with routine care, and the two formats did not differ significantly on eight of the ten symptoms. Both may therefore serve as adjunctive supportive care for lung cancer patients receiving chemotherapy. RV delivered on a standard screen or tablet does not require the equipment, cleaning and training time that a head-mounted display demands and may be a pragmatic and accessible option. The immersive format produced a further small gain in well-being, tiredness and distress and may be considered where these outcomes are prioritised, where the equipment and staff time can be accommodated or where the patient prefers it. Neither format should be regarded as a substitute for the pharmacological management of nausea or lack of appetite, for which no effect was found. Future research should extend follow-up beyond the initial treatment cycles, evaluate personalised and adaptive intervention content, examine predictors of treatment response, incorporate psychophysiological measures such as heart rate variability, assess cybersickness with a validated instrument, and assess the comparative cost-effectiveness of VR and RV in routine oncology care. Adequately powered comparative or non-inferiority trials are needed to determine whether either format is clinically preferable.

## 5. Conclusions

To our knowledge, this is the first three-arm randomised trial comparing immersive VR with a theme-matched RV in lung cancer patients receiving chemotherapy. Two sessions of immersive VR or RV reduced tiredness, depression, anxiety and acute distress and improved well-being compared with routine care. Drowsiness also decreased with both formats, and pain, shortness of breath and numbness with VR, although these differences were small. There was no evidence that the effect differed between the two cycles. Although adverse events were recorded by direct questioning rather than with a validated instrument, no serious adverse event was observed. Immersive delivery produced a further small improvement over RV in well-being, tiredness and distress, which for the ESAS symptoms remained below the minimal clinically important difference, and the trial was not designed to establish equivalence between the two formats. RV, which requires only a standard screen or tablet, may therefore be a useful and accessible supportive-care option. No intervention-specific effect emerged for nausea, lack of appetite or overall quality of life.

## Figures and Tables

**Table 1:** Baseline sociodemographic and clinical characteristics by group.

Characteristic	VR (*n* = 106)	RV (*n* = 104)	CG (*n* = 105)	χ^2^	*p*
**Sex**				0.11	0.947
Male, *n* (%)	83 (78.3%)	83 (79.8%)	82 (78.1%)		
Female, *n* (%)	23 (21.7%)	21 (20.2%)	23 (21.9%)		
**Age group**				2.42	0.659
<65 years, *n* (%)	51 (48.1%)	51 (49.0%)	42 (40.0%)		
65–74 years, *n* (%)	43 (40.6%)	39 (37.5%)	47 (44.8%)		
≥75 years, *n* (%)	12 (11.3%)	14 (13.5%)	16 (15.2%)		
**Marital status**				0.80	0.670
Single, *n* (%)	18 (17.0%)	15 (14.4%)	20 (19.0%)		
Married, *n* (%)	88 (83.0%)	89 (85.6%)	85 (81.0%)		
**Education**				6.48	0.594
Illiterate, *n* (%)	3 (2.8%)	7 (6.7%)	3 (2.9%)		
Primary, *n* (%)	43 (40.6%)	50 (48.1%)	46 (43.8%)		
Secondary, *n* (%)	19 (17.9%)	13 (12.5%)	20 (19.0%)		
High school, *n* (%)	24 (22.6%)	16 (15.4%)	18 (17.1%)		
University or higher, *n* (%)	17 (16.0%)	18 (17.3%)	18 (17.1%)		
**Lung cancer surgery**				0.59	0.744
Yes, *n* (%)	29 (27.4%)	27 (26.0%)	24 (22.9%)		
No, *n* (%)	77 (72.6%)	77 (74.0%)	81 (77.1%)		
**Radiotherapy**				0.75	0.688
Yes, *n* (%)	37 (34.9%)	31 (29.8%)	32 (30.5%)		
No, *n* (%)	69 (65.1%)	73 (70.2%)	73 (69.5%)		
**Chronic disease**				1.22	0.543
Yes, *n* (%)	78 (73.6%)	80 (76.9%)	84 (80.0%)		
No, *n* (%)	28 (26.4%)	24 (23.1%)	21 (20.0%)		
**Chemotherapy regimen**				1.60	0.999
Taxane, *n* (%)	32 (30.2%)	31 (29.8%)	28 (26.7%)		
Weekly/concurrent-RT taxane, *n* (%)	24 (22.6%)	23 (22.1%)	27 (25.7%)		
Etoposide, *n* (%)	25 (23.6%)	25 (24.0%)	25 (23.8%)		
Pemetrexed, *n* (%)	10 (9.4%)	9 (8.7%)	11 (10.5%)		
Gemcitabine, *n* (%)	8 (7.5%)	8 (7.7%)	9 (8.6%)		
Vinorelbine, *n* (%)	7 (6.6%)	8 (7.7%)	5 (4.8%)		
**Cancer stage**				0.07	0.968
Stage I–II/early/limited, *n* (%)	27 (25.5%)	28 (26.9%)	27 (25.7%)		
Stage III–IV/advanced/extensive, *n* (%)	79 (74.5%)	76 (73.1%)	78 (74.3%)		

Data are *n* (%). Between-group comparisons by Pearson χ^2^ test. RT, radiotherapy; VR, virtual reality; RV, relaxation video; CG, control group.

**Table 2:** Group × Time effects on within-session change, with the overall time effect and the Group × Cycle × Time interaction, for the ESAS symptoms (primary outcomes) and distress (secondary outcome).

Outcome	Time Main Effect			Group × Time					Group × Cycle × Time			
	*F*	*df*	*p*	*F*	*df*	*p*	*p*(*FDR*)	*f*	*F*	*df*	*p*	*f*
Pain	32.23	1, 231.2	<0.001 ***	5.90	2, 231.1	0.003 **	0.005 **	0.07	0.48	2, 215.1	0.620	0.02
Tiredness	54.46	1, 238.8	<0.001 ***	21.78	2, 238.7	<0.001 ***	<0.001 ***	0.12	0.27	2, 241.4	0.760	0.01
Nausea	3.77	1, 236.0	0.053	1.82	2, 235.9	0.164	0.182	0.05	2.84	2, 223.5	0.061	0.07
Depression	70.55	1, 296.9	<0.001 ***	20.50	2, 296.9	<0.001 ***	<0.001 ***	0.12	1.62	2, 275.1	0.201	0.03
Anxiety	92.91	1, 297.4	<0.001 ***	19.73	2, 297.4	<0.001 ***	<0.001 ***	0.12	1.99	2, 289.6	0.139	0.03
Drowsiness	21.93	1, 262.0	<0.001 ***	7.68	2, 262.0	<0.001 ***	0.001 **	0.04	1.55	2, 264.5	0.215	0.02
Lack of appetite	1.02	1, 210.7	0.313	0.60	2, 210.7	0.551	0.551	0.01	0.61	2, 210.3	0.543	0.01
Well-being	245.26	1, 286.0	<0.001 ***	61.60	2, 286.0	<0.001 ***	<0.001 ***	0.24	1.28	2, 254.9	0.280	0.03
Shortness of breath	4.83	1, 261.7	0.029 *	3.77	2, 261.7	0.024 *	0.035 *	0.03	1.27	2, 208.6	0.283	0.01
Numbness	9.01	1, 38.7	0.005 **	3.93	2, 38.7	0.028 *	0.035 *	0.05	0.09	2, 36.0	0.913	0.01
Distress (NCCN-DT)	144.05	1, 302.6	<0.001 ***	34.46	2, 302.6	<0.001 ***	−	0.17	0.61	2, 274.4	0.542	0.02

Linear mixed models (Section 2.7) fitted to all 1050 pre- and post-session assessments from the 525 sessions (intention-to-treat: VR *n* = 106, RV *n* = 104, CG *n* = 105); denominator degrees of freedom are Satterthwaite approximations. Time, within-session change across all groups; Group × Time, between-group difference in within-session change, averaged over the two cycles; Group × Cycle × Time, whether this difference varied between the cycles. *f*, Cohen’s *f* (Section 2.7; for Group × Cycle × Time, based on the between-cycle difference in change); 0.10, 0.25 and 0.40 denote small, medium and large effects. *p*(*FDR*), Benjamini–Hochberg-adjusted across the ten ESAS symptoms. * *p* < 0.05, ** *p* < 0.01, *** *p* < 0.001. ESAS, Edmonton Symptom Assessment Scale; NCCN-DT, National Comprehensive Cancer Network Distress Thermometer.

**Table 3:** Model-estimated within-session means and changes, and between-group differences in within-session change (Group × Time contrasts) for the ESAS symptoms (primary outcomes) and distress (secondary outcome).

Outcome	VR (*n* = 106)			RV (*n* = 104)			CG (*n* = 105)			Between-Group Differences in Within-Session Change (Group × Time)		
	Pre-EMM (SE)	Post-EMM (SE)	Change Δ (SE)	Pre-EMM (SE)	Post-EMM (SE)	Change Δ (SE)	Pre-EMM (SE)	Post-EMM (SE)	Change Δ (SE)	VR vs. CG: MD (95% CI) *d* (95% CI)	RV vs. CG: MD (95% CI) *d* (95% CI)	VR vs. RV: MD (95% CI) *d* (95% CI)
Pain	1.14 (0.19)	0.76 (0.17)	−0.39 ††† (0.07)	0.97 (0.19)	0.67 (0.17)	−0.29 ††† (0.07)	1.27 (0.19)	1.22 (0.17)	−0.04 (0.07)	MD = 0.35 (0.10, 0.60) ** d = 0.16 (0.04, 0.27)	MD = 0.25 (−0.001, 0.50) *d* = 0.11 (−0.001, 0.22)	MD = 0.10 (−0.15, 0.35) *d* = 0.04 (−0.07, 0.16)
Tiredness	2.90 (0.26)	2.06 (0.25)	−0.85 ††† (0.09)	3.11 (0.26)	2.74 (0.25)	−0.37 ††† (0.09)	2.89 (0.26)	2.92 (0.25)	0.03 (0.09)	MD = 0.87 (0.55, 1.19) *** d = 0.29 (0.18, 0.40)	MD = 0.40 (0.08, 0.71) ** d = 0.13 (0.03, 0.24)	MD = 0.47 (0.16, 0.79) ** d = 0.16 (0.05, 0.26)
Nausea	0.26 (0.09)	0.13 (0.09)	−0.14 †† (0.05)	0.24 (0.09)	0.23 (0.09)	−0.01 (0.05)	0.23 (0.09)	0.20 (0.09)	−0.03 (0.05)	MD = 0.11 (−0.06, 0.28) *d* = 0.10 (−0.06, 0.26)	MD = −0.02 (−0.19, 0.15) *d* = −0.02 (−0.18, 0.14)	MD = 0.13 (−0.05, 0.30) *d* = 0.12 (−0.04, 0.28)
Depression	2.20 (0.23)	1.47 (0.22)	−0.72 ††† (0.09)	2.03 (0.23)	1.48 (0.22)	−0.55 ††† (0.09)	2.41 (0.23)	2.43 (0.22)	0.02 (0.09)	MD = 0.74 (0.45, 1.04) *** d = 0.28 (0.17, 0.39)	MD = 0.58 (0.28, 0.87) *** d = 0.22 (0.11, 0.33)	MD = 0.17 (−0.13, 0.46) *d* = 0.06 (−0.05, 0.17)
Anxiety	2.16 (0.22)	1.37 (0.20)	−0.79 ††† (0.09)	2.04 (0.22)	1.43 (0.20)	−0.61 ††† (0.09)	2.36 (0.22)	2.31 (0.20)	−0.05 (0.09)	MD = 0.74 (0.45, 1.04) *** d = 0.27 (0.16, 0.38)	MD = 0.56 (0.26, 0.86) *** d = 0.20 (0.10, 0.31)	MD = 0.18 (−0.11, 0.48) *d* = 0.07 (−0.04, 0.18)
Drowsiness	2.63 (0.27)	2.37 (0.26)	−0.26 ††† (0.05)	2.17 (0.26)	2.01 (0.26)	−0.16 †† (0.05)	2.54 (0.26)	2.56 (0.26)	0.01 (0.05)	MD = 0.27 (0.10, 0.44) *** d = 0.09 (0.04, 0.15)	MD = 0.17 (0.002, 0.34) * d = 0.06 (0.001, 0.12)	MD = 0.10 (−0.07, 0.27) *d* = 0.04 (−0.02, 0.09)
Lack of appetite	1.45 (0.24)	1.43 (0.24)	−0.01 (0.05)	1.98 (0.24)	2.04 (0.24)	0.07 (0.05)	1.70 (0.24)	1.74 (0.24)	0.04 (0.05)	MD = 0.05 (−0.13, 0.23) *d* = 0.02 (−0.05, 0.09)	MD = −0.03 (−0.21, 0.15) *d* = −0.01 (−0.08, 0.05)	MD = 0.08 (−0.10, 0.26) *d* = 0.03 (−0.04, 0.10)
Well-being	3.31 (0.19)	2.00 (0.19)	−1.31 ††† (0.08)	2.94 (0.19)	2.12 (0.19)	−0.82 ††† (0.08)	3.18 (0.19)	3.12 (0.19)	−0.06 (0.08)	MD = 1.26 (0.98, 1.53) *** d = 0.59 (0.46, 0.72)	MD = 0.76 (0.49, 1.03) *** d = 0.36 (0.23, 0.49)	MD = 0.50 (0.22, 0.77) *** d = 0.23 (0.10, 0.36)
Shortness of breath	0.41 (0.14)	0.28 (0.13)	−0.13 ††† (0.04)	0.52 (0.14)	0.50 (0.13)	−0.02 (0.04)	0.76 (0.14)	0.77 (0.13)	0.01 (0.04)	MD = 0.14 (0.01, 0.27) * d = 0.08 (0.01, 0.15)	MD = 0.03 (−0.10, 0.16) *d* = 0.02 (−0.06, 0.09)	MD = 0.11 (−0.02, 0.23) *d* = 0.06 (−0.01, 0.13)
Numbness	0.56 (0.14)	0.40 (0.14)	−0.15 ††† (0.04)	0.55 (0.14)	0.49 (0.13)	−0.07 (0.04)	0.79 (0.14)	0.80 (0.13)	0.01 (0.04)	MD = 0.16 (0.02, 0.30) * d = 0.13 (0.01, 0.24)	MD = 0.07 (−0.07, 0.22) *d* = 0.06 (−0.05, 0.17)	MD = 0.09 (−0.06, 0.23) *d* = 0.07 (−0.04, 0.18)
Distress (NCCN-DT)	2.16 (0.23)	1.09 (0.22)	−1.08 ††† (0.09)	2.42 (0.23)	1.66 (0.21)	−0.77 ††† (0.09)	2.66 (0.23)	2.62 (0.21)	−0.04 (0.09)	MD = 1.04 (0.73, 1.34) *** d = 0.41 (0.29, 0.53)	MD = 0.73 (0.42, 1.03) *** d = 0.29 (0.17, 0.41)	MD = 0.31 (0.002, 0.62) * d = 0.12 (0.001, 0.24)

Estimated marginal means (EMM) and contrasts from the linear mixed model (Section 2.7), averaged over the two cycles (intention-to-treat; VR *n* = 106, RV *n* = 104, CG *n* = 105). Δ, post- minus pre-session change; lower scores indicate a better state on every ESAS item and on the NCCN-DT, so a negative Δ denotes improvement. MD, between-arm difference in within-session change (positive values favour the first-named arm); *d*, MD divided by the pooled baseline standard deviation (0.2, 0.5 and 0.8 denote small, medium and large effects). Confidence intervals and between-arm *p* values are Bonferroni-adjusted for the three pairwise contrasts; bounds close to zero are given to three decimals. †† *p* < 0.01, ††† *p* < 0.001 for within-group change (unadjusted); * *p* < 0.05, ** *p* < 0.01, *** *p* < 0.001 for between-arm differences. SE, standard error; VR, virtual reality; RV, relaxation video; CG, control group; NCCN-DT, National Comprehensive Cancer Network Distress Thermometer.

**Table 4:** EORTC QLQ-C30 quality-of-life outcomes: estimated means, within-group change and the Group × Time effect from baseline to the end of the second session.

EORTC QLQ-C30 Scale	VR (*n* = 106)			RV (*n* = 104)			CG (*n* = 105)			Time Main Effect			Group × Time			
	Baseline EMM (SE)	End EMM (SE)	Δ (SE), *p*	Baseline EMM (SE)	End EMM (SE)	Δ (SE), *p*	Baseline EMM (SE)	End EMM (SE)	Δ (SE), *p*	*F*	*df*	*p*	*F*	*df*	*p*	*f*
Global health status/QoL	59.36 (2.26)	66.93 (2.42)	+7.58 †† (2.64) *p* = 0.004	58.73 (2.28)	63.89 (2.35)	+5.15 † (2.58) *p* = 0.047	57.54 (2.27)	62.31 (2.36)	+4.77 (2.59) *p* = 0.067	15.05	1, 251.5	<0.001 ***	0.34	2, 251.4	0.715	0.05
EORTC QLQ-C30 Summary score	79.46 (1.70)	78.15 (1.88)	−1.31 (1.45) *p* = 0.370	76.85 (1.72)	77.90 (1.85)	+1.05 (1.41) *p* = 0.457	75.27 (1.71)	77.35 (1.85)	+2.08 (1.42) *p* = 0.144	0.54	1, 218.7	0.462	1.45	2, 218.7	0.236	0.08

Estimated marginal means (EMM) and SE at baseline (T1) and study end (T4) from group × time linear mixed models with an unstructured covariance matrix, using all available assessments (intention-to-treat; VR *n* = 106, RV *n* = 104, CG *n* = 105; 210 assessed at T4). Δ = T4 − T1. Scores range from 0 to 100; higher scores indicate better status. Time, change across all arms; Group × Time, between-arm difference in change; denominator degrees of freedom are Satterthwaite approximations; *f*, Cohen’s *f* as in Table 2. † *p* < 0.05, †† *p* < 0.01 for within-group change (unadjusted); *** *p* < 0.001 for Time and Group × Time. SE, standard error; VR, virtual reality; RV, relaxation video; CG, control group.

## Data Availability

The data presented in this study are available on request from the corresponding author due to conditions set by the Hacettepe University Ethics Committee of Clinical Trials regarding participant confidentiality.

## Supplementary Materials

The following supporting information can be downloaded at: https://www.mdpi.com/article/10.3390/healthcare14203394/s1, Table SA.1: Intervention content and assessment schedule; Table SA.2: Expert-panel content validity index (CVI); Table SA.3: Session procedure in the three groups; Table SB.1: Observed-data (non-imputed, unadjusted) descriptive statistics and mean comparisons; Table SB.2: Skin changes and mouth ulcer (additional items of the Turkish ESAS): scores and internal consistency; Table SB.3: Baseline comparison of participants who did and did not receive the second session; Table SC.1: Complete-case analysis: Group × Time effects on within-session change; Table SC.2: Cycle-specific analysis: Group × Time effects on within-session change; Figure SD.1: Estimated mean trajectories of well-being, tiredness, depression, anxiety and distress across the four assessments; Figure SD.2: Standardised between-arm differences in within-session change for the ESAS symptoms and distress. The completed CONSORT 2025 and TIDieR checklists are also provided.
