# Supplementary material for: Effects of Immersive Virtual Reality and Relaxation Video on Symptom Severity, Distress, and Quality of Life in Lung Cancer Patients During Initial Chemotherapy Cycles: A Three-Arm Randomised Controlled Trial
Source: Healthcare (Basel). 2026 Oct 12;14(20):3394. doi: 10.3390/healthcare14203394 (PMC13704489; doi:10.3390/healthcare14203394)
Supplement: Supplementary file 1 [file healthcare-14-03394-s001.zip › CONSORT_TIDieR_Checklists_v54.pdf]

Reporting Checklists

Effects of Immersive Virtual Reality and Relaxation Video on Symptom Severity, Distress, and Quality of Life in Lung Cancer Patients During Initial Chemotherapy Cycles: A Three-Arm Randomised Controlled Trial

1. CONSORT 2025 checklist

Section/topic	No	CONSORT 2025 checklist item description	Reported in (section)
**Title and abstract**			
Title and structured abstract	1a	Identification as a randomised trial	Title
	1b	Structured summary of the trial design, methods, results, and conclusions	Abstract
**Open science**			
Trial registration	2	Name of trial registry, identifying number (with URL) and date of registration	2.6 Ethical Considerations; Institutional Review Board Statement (ClinicalTrials.gov NCT06863623, https://clinicaltrials.gov/study/NCT06863623; first submitted 27 November 2024)
Protocol and statistical analysis plan	3	Where the trial protocol and statistical analysis plan can be accessed	Trial registry record (ClinicalTrials.gov NCT06863623), cited in 2.6 Ethical Considerations and the Institutional Review Board Statement
Data sharing	4	Where and how the individual de-identified participant data (including data dictionary), statistical code and any other materials can be accessed	Data Availability Statement
Funding and conflicts of interest	5a	Sources of funding and other support (eg, supply of drugs), and role of funders in the design, conduct, analysis and reporting of the trial	Funding
	5b	Financial and other conflicts of interest of the manuscript authors	Conflicts of Interest
**Introduction**			
Background and rationale	6	Scientific background and rationale	1. Introduction
Objectives	7	Specific objectives related to benefits and harms	1. Introduction
**Methods**			
Patient and public involvement	8	Details of patient or public involvement in the design, conduct and reporting of the trial	2.1 (pilot patient feedback); no formal patient or public involvement
Trial design	9	Description of trial design including type of trial (eg, parallel group, crossover), allocation ratio, and framework (eg, superiority, equivalence, non-inferiority, exploratory)	2.1 Design, Setting, and Participants
Changes to trial protocol	10	Important changes to the trial after it commenced including any outcomes or analyses that were not prespecified, with reason	2.7 Statistical Analysis
Trial setting	11	Settings (eg, community, hospital) and locations (eg, countries, sites) where the trial was conducted	2.1 Design, Setting, and Participants
Eligibility criteria	12a	Eligibility criteria for participants	2.1 Design, Setting, and Participants
	12b	If applicable, eligibility criteria for sites and for individuals delivering the interventions (eg, surgeons, physiotherapists)	2.4 Interventions
Intervention and comparator	13	Intervention and comparator with sufficient details to allow replication. If relevant, where additional materials describing the intervention and comparator (eg, intervention manual) can be accessed	2.4 Interventions; Figure 1; Suppl. Tables SA.1–SA.3
Outcomes	14	Prespecified primary and secondary outcomes, including the specific measurement variable (eg, systolic blood pressure), analysis metric (eg, change from baseline, final value, time to event), method of aggregation (eg, median, proportion), and time point for each outcome	2.5 Outcome Measures and Data Collection
Harms	15	How harms were defined and assessed (eg, systematically, non-systematically)	2.6 Ethical Considerations; 3.3 Adverse Events
Sample size	16a	How sample size was determined, including all assumptions supporting the sample size calculation	2.2 Sample Size Estimation
	16b	Explanation of any interim analyses and stopping guidelines	Not applicable; no interim analyses were performed
Randomisation:			
Sequence generation	17a	Who generated the random allocation sequence and the method used	2.3 Randomisation, Allocation, and Blinding
	17b	Type of randomisation and details of any restriction (eg, stratification, blocking and block size)	2.3 Randomisation, Allocation, and Blinding (stratified by cancer stage and chemotherapy protocol; restricted randomisation with a maximum tolerated imbalance of five rather than permuted blocks; not stratified by centre)
Allocation concealment mechanism	18	Mechanism used to implement the random allocation sequence (eg, central computer/telephone; sequentially numbered, opaque, sealed containers), describing any steps to conceal the sequence until interventions were	2.3 Randomisation, Allocation, and Blinding

		assigned	
Implementation	19	Whether the personnel who enrolled and those who assigned participants to the interventions had access to the random allocation sequence	2.3 Randomisation, Allocation, and Blinding
Blinding	20a	Who was blinded after assignment to interventions (eg, participants, care providers, outcome assessors, data analysts)	2.3 Randomisation, Allocation, and Blinding (open label; masked group codes during analysis)
	20b	If blinded, how blinding was achieved and description of the similarity of interventions	Not applicable (open label); similarity of the interventions: 2.4 Interventions
Statistical methods	21a	Statistical methods used to compare groups for primary and secondary outcomes, including harms	2.7 Statistical Analysis
	21b	Definition of who is included in each analysis (eg, all randomised participants), and in which group	2.7 Statistical Analysis
	21c	How missing data were handled in the analysis	2.7 Statistical Analysis (linear mixed model using all available assessments under a missing-at-random assumption); 3.1 Participant Flow and Baseline Characteristics; Figure 2; 4.1 Limitations; Suppl. Table SB.3
	21d	Methods for any additional analyses (eg, subgroup and sensitivity analyses), distinguishing prespecified from post hoc	2.7 Statistical Analysis (complete-case and cycle-specific sensitivity analyses, not prespecified); Suppl. Tables SC.1–SC.2
**Results**			
Participant flow, including flow diagram	22a	For each group, the numbers of participants who were randomly assigned, received intended intervention, and were analysed for the primary outcome	3.1 Participant Flow and Baseline Characteristics; Figure 2
	22b	For each group, losses and exclusions after randomisation, together with reasons	3.1 Participant Flow and Baseline Characteristics; Figure 2 (reasons for non-receipt of the second session by arm); Suppl. Table SB.3 (participants who did and did not receive the second session)
Recruitment	23a	Dates defining the periods of recruitment and follow-up for outcomes of benefits and harms	2.1 Design, Setting, and Participants (study period)
	23b	If relevant, why the trial ended or was stopped	Not applicable; the trial was not stopped early
Intervention and comparator delivery	24a	Intervention and comparator as they were actually administered (eg, where appropriate, who delivered the intervention/comparator, how participants adhered, whether they were delivered as intended (fidelity))	2.4 Interventions; Figure 1; Suppl. Tables SA.1 and SA.3; 3.1 Participant Flow and Baseline Characteristics; 3.3 Adverse Events; 4.1 Limitations (fidelity not independently scored)
	24b	Concomitant care received during the trial for each group	2.4 Interventions (routine care in all arms)
Baseline data	25	A table showing baseline demographic and clinical characteristics for each group	Table 1
Numbers analysed, outcomes and estimation	26	For each primary and secondary outcome, by group:<br>● the number of participants included in the analysis<br>● the number of participants with available data at the outcome time point<br>● result for each group, and the estimated effect size and its precision (such as 95% confidence interval)<br>● for binary outcomes, presentation of both absolute and relative effect size	3.2 Effects of Interventions; Tables 2–4; Suppl. Table SB.1
Harms	27	All harms or unintended events in each group	3.3 Adverse Events
Ancillary analyses	28	Any other analyses performed, including subgroup and sensitivity analyses, distinguishing pre-specified from post hoc	3.2.2 (complete-case and cycle-specific estimates); Suppl. Tables SC.1–SC.2
**Discussion**			
Interpretation	29	Interpretation consistent with results, balancing benefits and harms, and considering other relevant evidence	4. Discussion; 5. Conclusions
Limitations	30	Trial limitations, addressing sources of potential bias, imprecision, generalisability, and, if relevant, multiplicity of analyses	4.1 Limitations

Citation: Hopewell S, Chan AW, Collins GS, Hróbjartsson A, Moher D, Schulz KF, et al. CONSORT 2025 Statement: updated guideline for reporting randomised trials. BMJ. 2025; 389:e081123.
© 2025 Hopewell et al. This is an Open Access article distributed under the terms of the Creative Commons Attribution License (https://creativecommons.org/licenses/by/4.0/), which permits unrestricted use, distribution, and reproduction in any medium, provided the original work is properly cited. https://dx.doi.org/10.1136/bmj-2024-081123

*We strongly recommend reading this statement in conjunction with the CONSORT 2025 Explanation and Elaboration and/or the CONSORT 2025 Expanded Checklist for important clarifications on all the items. We also recommend reading relevant CONSORT extensions. See www.consort-spirit.org.

2. TIDieR (Template for Intervention Description and Replication)

TIDieR item	Description in this trial	Location
1. Brief name	Immersive VR session; theme-matched relaxation video (RV); routine care (control).	Title; 2.4
2. Why (rationale/theory)	Reduction of chemotherapy symptoms and distress burden via attentional distraction and a dampening of the physiological stress response.	1; 2.4
3. What (materials)	VR: Meta Quest 2 head-mounted display + hand controllers; nature scenes, with nature sounds and background music, from the Greener Games Nature Treks VR application. RV: ≥12.4-inch screen + Bluetooth headphones; slide-video built from royalty-free Pixabay images with relaxing background music and ambient sounds from YouTube. Content reviewed and approved by a five-expert panel (content validity in Suppl. Table SA.2).	2.4; Suppl. Table SA.2
4. What (procedures)	Nature-based themes (Forest and Beach): session 1 "Walking in the Forest" (Green Meadows), session 2 "Sitting on the Beach" (Blue Ocean). Both delivered at least 30 minutes after infusion initiation to allow monitoring of acute infusion-related reactions. The head-mounted display, hand controllers and headphones were disinfected with 70% alcohol before and after each use, and single-use pads were applied to the headphones.	2.4; Suppl. Tables SA.1 and SA.3
5. Who provided	A single trained researcher across the three centres (for procedural consistency); fidelity not independently assessed.	2.4; 4.1
6. How (mode of delivery)	Individual, face-to-face; VR immersive, RV non-immersive audiovisual.	2.4
7. Where (setting)	Outpatient chemotherapy units of three oncology hospitals in Ankara, Türkiye.	2.1
8. When and how much (dose)	20 minutes per session; one session during each of the first two chemotherapy cycles (two sessions total).	2.4
9. Tailoring	None; content and sequence standardised to preserve internal validity.	2.4; 4.1
10. Modifications	Refined during pilot testing with 10 lung cancer patients (not included in the main sample): the 20-minute session duration and the two-cycle delivery schedule were set, and the two scenarios delivered (Walking in the Forest, Sitting on the Beach) were selected from six candidate nature environments.	2.1
11. How well (planned fidelity)	Single provider for procedural consistency; expert-panel content validity (Suppl. Table SA.2); no independent fidelity scoring.	2.4; 4.1; Suppl. Table SA.2
12. How well (actual fidelity)	Adverse events and patient experience were recorded on the Patient Follow-up Form; all randomised participants completed the first session, and no adverse event required discontinuation of a session.	2.5; 3.1; 3.3
