# Supplementary material for: Effects of Immersive Virtual Reality and Relaxation Video on Symptom Severity, Distress, and Quality of Life in Lung Cancer Patients During Initial Chemotherapy Cycles: A Three-Arm Randomised Controlled Trial
Source: Healthcare (Basel). 2026 Oct 12;14(20):3394. doi: 10.3390/healthcare14203394 (PMC13704489; doi:10.3390/healthcare14203394)
Supplement: Supplementary file 1 [file healthcare-14-03394-s001.zip › Supplementary Material_v54.pdf]

Supplementary Materials

Effects of Immersive Virtual Reality and Relaxation Video on Symptom Severity, Distress, and Quality of Life in Lung Cancer Patients During Initial Chemotherapy Cycles: A Three-Arm Randomised Controlled Trial

Supplementary Section A: Intervention Content, Content Validity and Session Procedure

Table SA.1. Intervention content and assessment schedule

Session	VR group			RV group	
Session 1	Walking in the Forest (Greener Games Nature Treks VR, Green Meadows)			Walking in the Forest themed audiovisual sequence	
Session 2	Sitting on the Beach (Greener Games Nature Treks VR, Blue Ocean)			Sitting on the Beach themed audiovisual sequence	
			Assessment schedule		
**Time point**	**PIF**	**PFF**	**ESAS**	**NCCN-DT**	**EORTC**
Pre-intervention (T1, cycle 1)	✓	✓	✓	✓	✓
Post-intervention (T2, cycle 1)	—	✓	✓	✓	—
Pre-intervention (T3, cycle 2)	—	✓	✓	✓	—
Post-intervention (T4, cycle 2)	—	✓	✓	✓	✓

Note. PIF, Patient Information Form; PFF, Patient Follow-up Form; ESAS, Edmonton Symptom Assessment Scale; NCCN-DT, NCCN Distress Thermometer; EORTC, EORTC QLQ-C30. EORTC QLQ-C30 was administered at T1 and T4 only (reflecting the preceding week). VR, virtual reality; RV, relaxation video; —, not administered.

Table SA.2. Expert-panel content validity index (CVI)

Criterion / item	Experts (*n*)	I-CVI Walking in the Forest (session 1)	I-CVI Sitting on the Beach (session 2)	All six candidate themes, I-CVI range
Distraction (VR themes)	5	1.00 (5/5)	1.00 (5/5)	1.00 (all six themes)
Relaxation (VR themes)	5	1.00 (5/5)	1.00 (5/5)	0.80–1.00 (Island Discovery: 0.80, Ocean Discovery: 0.80, Others: 1.00)
Distraction (matched relaxation videos)	5	0.80 (4/5)	0.80 (4/5)	0.80 (all six themes)
Relaxation (matched relaxation videos)	5	1.00 (5/5)	1.00 (5/5)	0.80–1.00 (Savanna Tour: 0.80, Others: 1.00)
RV–VR theme matching (theme appropriateness of videos)	5	0.80 (4/5)	0.80 (4/5)	0.60–1.00 (Savanna Tour: 0.60, Island Discovery: 0.80, Walking in the Forest: 0.80, Swimming in the Sea: 0.80, Sitting on the Beach: 0.80, Ocean Discovery: 1.00)

Note. Appropriateness of the candidate scenes was rated by a five-expert panel (0 = no, 1 = yes). Parentheses show the number of agreeing experts (*n*/5). Overall S-CVI/Ave was 0.90. Following the expert ratings, the experts' qualitative comments and pilot feedback, "Walking in the Forest" and "Sitting on the Beach" were chosen as the primary trial scenes (S-CVI/Ave = 0.92). I-CVI, item-level content validity index; S-CVI/Ave, scale-level content validity index (average of the I-CVIs); VR, virtual reality; RV, relaxation video.

Table SA.3. Session procedure in the three groups

Step	Timing	VR group	RV group	Control group
Preparation	Before the first enrolment	Pilot testing with 10 patients who were not part of the trial sample helped determine the session duration (20 minutes), the two-cycle schedule and the two scenes (Walking in the Forest, Sitting on the Beach), which were selected from six candidate nature environments. The relaxation videos were built as slide videos from royalty-free Pixabay images with relaxing background music and ambient sounds obtained from YouTube, matched to the VR scenes in theme, sequence and duration, with no narration or breathing instruction. Both interventions were reviewed by a five-expert panel (Table SA.2). The researcher completed the device training and rehearsed the full session protocol, including the safety checks.		
Enrolment	First chemotherapy day of cycle 1	Eligibility confirmed; written informed consent; Patient Information Form (sociodemographic and clinical characteristics).		
Allocation	After enrolment	Computer-generated sequence (R), stratified by cancer stage and chemotherapy protocol, 1:1:1; the allocation was obtained by telephone from a researcher not involved in intervention delivery or data collection.		
Pre-session assessment (T1)	Cycle 1, immediately before session 1	ESAS, NCCN-DT and EORTC QLQ-C30.		
Preparation of session 1	At least 30 minutes after infusion initiation, generally in the middle phase of the infusion; immediately after T1	Meta Quest 2 head-mounted display and hand controllers disinfected with 70% alcohol and fitted; audio level and position adjusted for comfort; participant told that the session could be stopped at any point.	Screen of at least 12.4 inches; Bluetooth headphones disinfected with 70% alcohol and fitted with single-use pads; audio level and position adjusted for comfort; participant told that the session could be stopped at any point.	Routine care alone, including monitoring of vital signs and chemotherapy-related side effects.
Session 1 (20 minutes)	Cycle 1	Walking in the Forest (Greener Games Nature Treks VR, Green Meadows); the participant looks around freely and moves with the hand controllers; the researcher does not intervene and observes throughout.	Walking in the Forest themed video, non-immersive; the researcher does not intervene and observes throughout.	Routine care alone over the same 20 minutes, with no additional distraction or supportive intervention.
Post-session assessment (T2)	Immediately after session 1	ESAS and NCCN-DT (in the CG, 20 minutes after T1). Participants were asked whether they had experienced any problem, such as cybersickness symptoms, general discomfort or an infusion-related reaction; any reported problem was recorded on the Patient Follow-up Form.		
Post-session device hygiene	After session 1	Head-mounted display and controllers disinfected again with 70% alcohol.	Headphones disinfected again with 70% alcohol.	–
Pre-session assessment (T3)	Cycle 2, next chemotherapy cycle, immediately before session 2	ESAS and NCCN-DT.		
Preparation of session 2	At least 30 minutes after infusion initiation, generally in the middle phase of the infusion; immediately after T3	Meta Quest 2 head-mounted display and hand controllers disinfected with 70% alcohol and fitted; audio level and position adjusted for comfort; participant told that the session could be stopped at any point.	Screen of at least 12.4 inches; Bluetooth headphones disinfected with 70% alcohol and fitted with single-use pads; audio level and position adjusted for comfort; participant told that the session could be stopped at any point.	Routine care alone, including monitoring of vital signs and chemotherapy-related side effects.
Session 2 (20 minutes)	Cycle 2, next chemotherapy cycle	Sitting on the Beach (Greener Games Nature Treks VR, Blue Ocean); the participant looks around freely and moves with the hand controllers; the researcher does not intervene and observes throughout.	Sitting on the Beach themed video, non-immersive; the researcher does not intervene and observes throughout.	Routine care alone over the same 20 minutes, with no additional distraction or supportive intervention.
Post-session assessment (T4)	Immediately after session 2	ESAS, NCCN-DT and EORTC QLQ-C30 (in the CG, 20 minutes after T3). Participants were asked whether they had experienced any problem, such as cybersickness symptoms, general discomfort or an infusion-related reaction; any reported problem was recorded on the Patient Follow-up Form.		
Post-session device hygiene	After session 2	Head-mounted display and controllers disinfected again with 70% alcohol.	Headphones disinfected again with 70% alcohol.	–

Note. T1–T4 as in Table SA.1; the content of each session is given in Table SA.1 and the expert-panel review in Table SA.2. All sessions were delivered by the same researcher at the three centres. ESAS, Edmonton Symptom Assessment Scale; NCCN-DT, NCCN Distress Thermometer; EORTC QLQ-C30, European Organization for Research and Treatment of Cancer Quality of Life Questionnaire-Core 30; VR, virtual reality; RV, relaxation video; CG, control group; –, not applicable.

Supplementary Section B: Observed-Data Analyses

Table SB.1. Observed-data (non-imputed, unadjusted) descriptive statistics and mean comparisons

Outcome / time	Cycle 1 (T1 pre – T2 post)					Cycle 2 (T3 pre – T4 post)				
	VR	RV	CG	*F*	*p*	VR	RV	CG	*F*	*p*
Pain, pre-	1.28 ± 2.49	1.16 ± 1.91	1.22 ± 2.25	0.08	.927	0.93 ± 1.92	0.78 ± 1.83	1.28 ± 2.58	1.05	.353
Pain, post-	0.83 ± 1.85	0.75 ± 1.74	1.13 ± 2.21	1.13	.325	0.63 ± 1.59	0.61 ± 1.68	1.30 ± 2.46	2.82	.062
**Pain, within-group paired *t***	*t*=−4.09***	*t*=−4.39***	*t*=−1.58	—	—	*t*=−2.41*	*t*=−2.11*	*t*=0.15	—	—
Tiredness, pre-	2.88 ± 2.77	3.02 ± 3.06	2.75 ± 3.14	0.21	.813	2.85 ± 2.82	3.19 ± 3.01	3.03 ± 2.95	0.24	.788
Tiredness, post-	1.90 ± 2.38	2.60 ± 3.02	2.73 ± 3.16	2.58	.078	2.13 ± 2.40	2.88 ± 2.91	3.10 ± 2.98	2.25	.108
**Tiredness, within-group paired *t***	*t*=−6.08***	*t*=−3.29**	*t*=−0.63	—	—	*t*=−3.68***	*t*=−2.02*	*t*=1.69	—	—
Nausea, pre-	0.32 ± 1.44	0.17 ± 1.05	0.12 ± 0.55	0.96	.385	0.21 ± 0.66	0.22 ± 1.04	0.35 ± 1.32	0.40	.671
Nausea, post-	0.12 ± 0.64	0.28 ± 1.42	0.10 ± 0.53	1.14	.322	0.13 ± 0.52	0.11 ± 0.83	0.32 ± 1.31	1.07	.345
**Nausea, within-group paired *t***	*t*=−1.69	*t*=1.10	*t*=−1.75	—	—	*t*=−1.40	*t*=−1.53	*t*=−1.00	—	—
Depression, pre-	2.35 ± 2.72	1.98 ± 2.55	2.72 ± 2.70	2.04	.131	2.10 ± 2.75	2.14 ± 2.40	2.24 ± 2.66	0.05	.951
Depression, post-	1.42 ± 2.04	1.38 ± 2.26	2.69 ± 2.68	10.50	<.001<br>CG>VR***; CG>RV***	1.57 ± 2.44	1.63 ± 2.33	2.32 ± 2.68	2.01	.136
**Depression, within-group paired *t***	*t*=−5.67***	*t*=−5.09***	*t*=−1.65	—	—	*t*=−4.67***	*t*=−4.27***	*t*=1.18	—	—
Anxiety, pre-	2.33 ± 2.61	2.42 ± 2.61	2.83 ± 2.97	0.99	.374	2.01 ± 2.60	1.68 ± 2.06	1.92 ± 2.50	0.36	.697
Anxiety, post-	1.29 ± 1.95	1.51 ± 2.28	2.69 ± 2.89	10.25	<.001<br>CG>VR***; CG>RV**	1.46 ± 2.22	1.38 ± 1.97	1.96 ± 2.47	1.41	.247
**Anxiety, within-group paired *t***	*t*=−5.84***	*t*=−6.58***	*t*=−2.69**	—	—	*t*=−3.95***	*t*=−3.91***	*t*=0.73	—	—
Drowsiness, pre-	2.50 ± 2.97	2.05 ± 2.71	2.14 ± 2.91	0.73	.483	2.70 ± 3.23	2.22 ± 2.80	3.00 ± 3.31	1.13	.325
Drowsiness, post-	2.12 ± 2.87	1.89 ± 2.58	2.12 ± 2.89	0.24	.790	2.57 ± 3.27	2.07 ± 2.79	3.04 ± 3.37	1.71	.184
**Drowsiness, within-group paired *t***	*t*=−3.31**	*t*=−3.15**	*t*=−0.50	—	—	*t*=−2.12*	*t*=−1.84	*t*=1.00	—	—
Lack of appetite, pre-	1.29 ± 2.37	1.89 ± 2.96	1.98 ± 2.84	1.99	.139	1.52 ± 2.40	2.08 ± 3.34	1.49 ± 2.52	1.01	.366
Lack of appetite, post-	1.26 ± 2.32	1.88 ± 2.98	1.93 ± 2.83	1.96	.143	1.52 ± 2.37	2.24 ± 3.36	1.62 ± 2.71	1.30	.275
**Lack of appetite, within-group paired *t***	*t*=−1.14	*t*=−0.47	*t*=−1.00	—	—	*t*=0.00	*t*=1.56	*t*=1.00	—	—
Well-being, pre-	3.17 ± 2.12	2.99 ± 2.02	3.10 ± 2.24	0.19	.827	3.40 ± 2.26	2.89 ± 1.90	3.31 ± 2.23	1.16	.315
Well-being, post-	1.71 ± 1.89	2.07 ± 2.12	3.05 ± 2.23	11.64	<.001<br>CG>VR***; CG>RV**	2.22 ± 2.32	2.18 ± 1.84	3.25 ± 2.15	5.86	.003<br>CG>VR*; CG>RV**
**Well-being, within-group paired *t***	*t*=−11.68***	*t*=−9.40***	*t*=−1.28	—	—	*t*=−8.09***	*t*=−5.85***	*t*=−0.85	—	—
Shortness of breath, pre-	0.44 ± 1.37	0.56 ± 1.65	0.86 ± 2.13	1.58	.207	0.33 ± 1.06	0.53 ± 1.31	0.49 ± 1.56	0.44	.645
Shortness of breath, post-	0.36 ± 1.03	0.54 ± 1.62	0.87 ± 2.14	2.55	.080	0.18 ± 0.60	0.53 ± 1.31	0.51 ± 1.56	1.72	.182
**Shortness of breath, within-group paired *t***	*t*=−1.90	*t*=−0.63	*t*=1.00	—	—	*t*=−1.56	—	*t*=1.00	—	—
Numbness, pre-	0.25 ± 1.01	0.31 ± 1.18	0.64 ± 1.60	2.74	.066	0.79 ± 1.85	0.81 ± 2.09	0.90 ± 2.06	0.06	.940
Numbness, post-	0.11 ± 0.44	0.26 ± 1.16	0.64 ± 1.60	5.67	.004<br>CG>VR**	0.61 ± 1.67	0.72 ± 2.00	0.92 ± 2.09	0.44	.646
**Numbness, within-group paired *t***	*t*=−1.58	*t*=−2.28*	—	—	—	*t*=−2.34*	*t*=−1.00	*t*=1.00	—	—
Distress (NCCN-DT), pre-	2.22 ± 2.22	2.46 ± 2.54	2.70 ± 2.79	0.95	.390	2.25 ± 2.46	2.31 ± 2.65	2.65 ± 2.67	0.48	.622
Distress (NCCN-DT), post-	1.02 ± 1.51	1.50 ± 2.27	2.56 ± 2.73	13.27	<.001<br>CG>VR***; CG>RV**	1.25 ± 1.98	1.75 ± 2.60	2.69 ± 2.73	6.06	.003<br>CG>VR**
**Distress (NCCN-DT), within-group paired *t***	*t*=−8.32***	*t*=−7.38***	*t*=−1.89	—	—	*t*=−6.79***	*t*=−4.82***	*t*=1.00	—	—
**EORTC QLQ-C30 quality of life (observed; Cycle 1 block = baseline T1, Cycle 2 block = study end T4)**										
Global health status/QoL	59.36 ± 23.73	58.73 ± 23.26	57.54 ± 22.90	0.17	.848	67.16 ± 20.46	62.96 ± 21.62	63.26 ± 19.85	0.88	.416
**Global health status/QoL within-group paired *t***	—	—	—	—	—	*t*=2.56*	*t*=2.31*	*t*=1.19	—	—
EORTC QLQ-C30 Summary score	79.46 ± 16.94	76.85 ± 16.33	75.27 ± 19.19	1.53	.217	79.29 ± 17.75	78.21 ± 17.29	78.56 ± 16.13	0.07	.931
**EORTC QLQ-C30 Summary score within-group paired *t***	—	—	—	—	—	*t*=−1.04	*t*=0.64	*t*=1.33	—	—

Note. Observed data; no imputation. VR/RV/CG *n* = 106/104/105 at T1 and T2 and 67/72/71 at T3 and T4. Mean ± SD by group. *F*/p, between-group one-way ANOVA at that time point, with significant Bonferroni-adjusted pairwise contrasts. Within-group paired *t*: T2 − T1 and T4 − T3 for ESAS and NCCN-DT, T4 − T1 for EORTC QLQ-C30. Higher ESAS/NCCN-DT scores are worse; higher EORTC QLQ-C30 scores are better. **p*<.05, ***p*<.01, ****p*<.001. ESAS, Edmonton Symptom Assessment Scale; NCCN-DT, National Comprehensive Cancer Network Distress Thermometer; QoL, quality of life; SD, standard deviation; VR, virtual reality; RV, relaxation video; CG, control group; —, not applicable or no within-group variation.

Table SB.2. Skin changes and mouth ulcer (additional items of the Turkish ESAS): scores and internal consistency

Item	T1 mean (SD)	T2 mean (SD)	T3 mean (SD)	T4 mean (SD)	Reported at T1, *n* (%)	Reported at T3, *n* (%)	Sessions with any change
**Scores at the four assessments**							
Skin changes	0.00 (0.00)	0.00 (0.00)	0.04 (0.32)	0.04 (0.32)	0 (0.0%)	3 (1.4%)	0 of 525
Mouth ulcer	0.12 (0.72)	0.12 (0.72)	0.13 (0.59)	0.13 (0.59)	13 (4.1%)	12 (5.7%)	0 of 525

Internal consistency

ESAS items	Cronbach's alpha			
	T1	T2	T3	T4
Ten symptoms analysed	0.69	0.70	0.71	0.73
All twelve items of the Turkish adaptation	0.68	0.69	0.70	0.72

Note. Both items were administered at all four assessments (n = 315 at T1 and T2; n = 210 at T3 and T4). Neither changed between the pre- and post-session assessments in any of the 525 sessions, so no within-session estimate could be obtained and they were not included in the inferential analyses. Internal consistency was marginally higher without the two items at every assessment. T1 and T3, before the first and second sessions; T2 and T4, after them. ESAS, Edmonton Symptom Assessment Scale; SD, standard deviation.

Table SB.3. Baseline comparison of participants who did and did not receive the second session

Baseline measure (T1)	*n* assessed	Mean ± SD assessed	*n* not assessed	Mean ± SD not assessed	Standardised difference	*p*
Pain	210	1.11 ± 2.02	105	1.44 ± 2.58	−0.15	.262
Tiredness	210	2.79 ± 2.96	105	3.07 ± 3.04	−0.09	.444
Nausea	210	0.21 ± 1.08	105	0.19 ± 1.07	0.02	.853
Depression	210	2.54 ± 2.77	105	1.98 ± 2.43	0.21	.068
Anxiety	210	2.62 ± 2.85	105	2.34 ± 2.49	0.10	.378
Drowsiness	210	2.18 ± 2.80	105	2.34 ± 3.00	−0.06	.635
Lack of appetite	210	1.72 ± 2.66	105	1.71 ± 2.90	0.00	.978
Well-being	210	3.10 ± 2.18	105	3.06 ± 2.02	0.02	.848
Shortness of breath	210	0.50 ± 1.61	105	0.85 ± 1.99	−0.20	.127
Numbness	210	0.29 ± 1.00	105	0.62 ± 1.73	−0.26	.073
Distress (NCCN-DT)	210	2.54 ± 2.49	105	2.30 ± 2.59	0.10	.429
Global health status/QoL	210	58.73 ± 23.88	105	58.17 ± 22.02	0.02	.838
QLQ-C30 summary score	210	78.33 ± 17.11	105	74.95 ± 18.32	0.19	.116

Note. Welch tests on the baseline (T1) values. Standardised difference, difference in means (assessed − not assessed) divided by the pooled SD. The proportion assessed at the second session did not differ across arms (χ^2 = 0.92, *p* = .631). NCCN-DT, National Comprehensive Cancer Network Distress Thermometer; QoL, quality of life; SD, standard deviation.

Table SB.3 (continued). Within-session change in the first session by receipt of the second session

Within-session change in the first session (T2 − T1)	Mean ± SD, received the second session (*n* = 210)	Mean ± SD, did not receive it (*n* = 105)	*p*, all	*p*, VR	*p*, RV	*p*, CG
Pain	−0.26 ± 0.76	−0.44 ± 1.19	.159	.240	.882	.272
Tiredness	−0.39 ± 1.14	−0.66 ± 1.54	.112	.081	.809	.260
Nausea	−0.08 ± 0.76	0.04 ± 1.16	.341	.506	.347	.308
Depression	−0.55 ± 1.27	−0.47 ± 1.21	.583	.473	.833	.766
Anxiety	−0.69 ± 1.43	−0.72 ± 1.43	.824	.780	.800	.510
Drowsiness	−0.18 ± 0.72	−0.19 ± 0.90	.925	.841	.021	.206
Lack of appetite	−0.01 ± 0.33	−0.07 ± 0.51	.337	.461	.641	.325
Well-being	−0.82 ± 1.14	−0.81 ± 1.13	.944	.873	.920	.072
Shortness of breath	−0.01 ± 0.28	−0.07 ± 0.40	.231	.442	.264	.325
Numbness	−0.02 ± 0.14	−0.15 ± 0.93	.146	.160	.676	–
Distress (NCCN-DT)	−0.80 ± 1.34	−0.69 ± 1.22	.431	.141	.610	.320

Note. Change is the post-session minus the pre-session score, so a negative value denotes improvement. Welch tests comparing participants who did and did not receive the second session, in the whole sample and within each arm; –, no variation in either subgroup. VR, virtual reality; RV, relaxation video; CG, control group.

Supplementary Section C: Sensitivity Analyses

Table SC.1. Complete-case analysis: Group × Time effects on within-session change

Outcome	Time main effect			Group × Time					Group × Cycle × Time			
	F	*df*	*p*	*F*	*df*	*p*	*p(FDR)*	*f*	*F*	*df*	*p*	*f*
Pain	23.22	1, 207.0	<.001***	5.49	2, 207.0	.005**	.008**	0.06	0.88	2, 207.0	.417	0.02
Tiredness	40.76	1, 207.0	<.001***	15.81	2, 207.0	<.001***	<.001***	0.11	0.06	2, 207.0	.941	0.01
Nausea	6.52	1, 207.0	.011*	2.02	2, 207.0	.136	.151	0.06	2.13	2, 207.0	.121	0.06
Depression	57.44	1, 207.0	<.001***	16.85	2, 207.0	<.001***	<.001***	0.13	1.85	2, 207.0	.160	0.03
Anxiety	68.20	1, 207.0	<.001***	15.73	2, 207.0	<.001***	<.001***	0.12	1.73	2, 207.0	.180	0.03
Drowsiness	19.04	1, 207.0	<.001***	7.36	2, 207.0	<.001***	.002**	0.04	0.93	2, 207.0	.397	0.02
Lack of appetite	1.68	1, 207.0	.196	0.58	2, 207.0	.559	.559	0.01	0.64	2, 207.0	.529	0.01
Well-being	193.33	1, 207.0	<.001***	46.10	2, 207.0	<.001***	<.001***	0.24	0.80	2, 207.0	.450	0.02
Shortness of breath	1.77	1, 207.0	.184	2.64	2, 207.0	.074	.106	0.03	1.49	2, 207.0	.229	0.01
Numbness	6.87	1, 207.0	.009**	2.47	2, 207.0	.087	.108	0.03	1.76	2, 207.0	.175	0.03
Distress (NCCN-DT)	113.93	1, 207.0	<.001***	27.29	2, 207.0	<.001***	–	0.18	0.32	2, 207.0	.727	0.01

Note. F, df and p from the linear mixed model described in Section 2.7, refitted to the 840 pre- and post-session assessments of the 210 participants who provided all four assessments (VR n = 67, RV n = 72, CG n = 71). Time, Group × Time, Group × Cycle × Time, f and $p(FDR)$ are defined as in main-text Table 2; f uses the pooled baseline standard deviation of the full randomised sample, so that its values can be compared with Table 2. Denominator degrees of freedom are Satterthwaite approximations. *p<.05, **p<.01, ***p<.001. NCCN-DT, National Comprehensive Cancer Network Distress Thermometer.

Table SC.1 (continued). Complete-case analysis: model-estimated within-session means and changes, and between-group differences in within-session change

Outcome	VR (n = 67)			RV (n = 72)			CG (n = 71)			Between-group differences in within-session change (Group × Time)		
	Pre-EMM (SE)	Post-EMM (SE)	Change Δ (SE)	Pre-EMM (SE)	Post-EMM (SE)	Change Δ (SE)	Pre-EMM (SE)	Post-EMM (SE)	Change Δ (SE)	VR vs CG: MD (95% CI) *d* (95% CI)	RV vs CG: MD (95% CI) *d* (95% CI)	VR vs RV: MD (95% CI) *d* (95% CI)
Pain	0.98 (0.21)	0.66 (0.19)	−0.32††† (0.07)	0.99 (0.20)	0.70 (0.18)	−0.28††† (0.07)	1.20 (0.20)	1.19 (0.18)	−0.01 (0.07)	MD = 0.31 (0.06, 0.57)**<br>*d* = 0.14 (0.03, 0.25)	MD = 0.28 (0.03, 0.53)*<br>*d* = 0.12 (0.01, 0.24)	MD = 0.04 (−0.22, 0.29)<br>*d* = 0.02 (−0.10, 0.13)
Tiredness	2.72 (0.31)	1.99 (0.30)	−0.73††† (0.10)	3.11 (0.30)	2.73 (0.29)	−0.38††† (0.10)	2.88 (0.30)	2.92 (0.29)	0.04 (0.10)	MD = 0.77 (0.44, 1.11)***<br>*d* = 0.26 (0.15, 0.37)	MD = 0.42 (0.10, 0.75)**<br>*d* = 0.14 (0.03, 0.25)	MD = 0.35 (0.02, 0.68)*<br>*d* = 0.12 (0.01, 0.23)
Nausea	0.30 (0.10)	0.13 (0.08)	−0.16†† (0.05)	0.17 (0.10)	0.13 (0.08)	−0.05 (0.05)	0.25 (0.10)	0.23 (0.08)	−0.02 (0.05)	MD = 0.14 (−0.04, 0.32)<br>*d* = 0.13 (−0.04, 0.30)	MD = 0.03 (−0.15, 0.21)<br>*d* = 0.03 (−0.14, 0.19)	MD = 0.12 (−0.07, 0.30)<br>*d* = 0.11 (−0.06, 0.28)
Depression	2.31 (0.28)	1.53 (0.26)	−0.78††† (0.10)	2.12 (0.27)	1.56 (0.25)	−0.56††† (0.10)	2.63 (0.28)	2.65 (0.25)	0.02 (0.10)	MD = 0.80 (0.45, 1.14)***<br>*d* = 0.30 (0.17, 0.43)	MD = 0.58 (0.24, 0.92)***<br>*d* = 0.22 (0.09, 0.35)	MD = 0.21 (−0.13, 0.56)<br>*d* = 0.08 (−0.05, 0.21)
Anxiety	2.22 (0.26)	1.40 (0.24)	−0.81††† (0.10)	2.09 (0.25)	1.49 (0.23)	−0.60††† (0.10)	2.42 (0.26)	2.39 (0.23)	−0.04 (0.10)	MD = 0.78 (0.43, 1.13)***<br>*d* = 0.28 (0.16, 0.41)	MD = 0.56 (0.22, 0.90)***<br>*d* = 0.21 (0.08, 0.33)	MD = 0.22 (−0.13, 0.56)<br>*d* = 0.08 (−0.05, 0.21)
Drowsiness	2.53 (0.30)	2.28 (0.30)	−0.25††† (0.05)	2.08 (0.29)	1.90 (0.29)	−0.18††† (0.05)	2.63 (0.29)	2.65 (0.29)	0.03 (0.05)	MD = 0.27 (0.09, 0.46)***<br>*d* = 0.10 (0.03, 0.16)	MD = 0.21 (0.03, 0.39)*<br>*d* = 0.07 (0.01, 0.14)	MD = 0.07 (−0.12, 0.25)<br>*d* = 0.02 (−0.04, 0.09)
Lack of appetite	1.28 (0.27)	1.28 (0.28)	−0.01 (0.05)	2.03 (0.26)	2.10 (0.27)	0.06 (0.05)	1.80 (0.26)	1.86 (0.27)	0.06 (0.05)	MD = 0.07 (−0.11, 0.25)<br>*d* = 0.03 (−0.04, 0.09)	MD = 0.00 (−0.18, 0.18)<br>*d* = 0.00 (−0.06, 0.07)	MD = 0.07 (−0.11, 0.25)<br>*d* = 0.03 (−0.04, 0.09)
Well-being	3.23 (0.22)	1.90 (0.23)	−1.33††† (0.09)	2.94 (0.22)	2.13 (0.22)	−0.81††† (0.09)	3.28 (0.22)	3.20 (0.22)	−0.08 (0.09)	MD = 1.25 (0.93, 1.57)***<br>*d* = 0.59 (0.44, 0.74)	MD = 0.74 (0.42, 1.05)***<br>*d* = 0.35 (0.20, 0.49)	MD = 0.52 (0.20, 0.83)***<br>*d* = 0.24 (0.09, 0.39)
Shortness of breath	0.34 (0.16)	0.24 (0.15)	−0.10†† (0.04)	0.58 (0.15)	0.59 (0.14)	0.01 (0.04)	0.50 (0.16)	0.51 (0.14)	0.01 (0.04)	MD = 0.11 (−0.02, 0.25)<br>*d* = 0.06 (−0.01, 0.14)	MD = 0.00 (−0.13, 0.13)<br>*d* = 0.00 (−0.08, 0.08)	MD = 0.11 (−0.02, 0.25)<br>*d* = 0.06 (−0.01, 0.14)
Numbness	0.43 (0.14)	0.34 (0.14)	−0.10†† (0.03)	0.57 (0.14)	0.51 (0.13)	−0.06 (0.03)	0.68 (0.14)	0.68 (0.13)	0.01 (0.03)	MD = 0.10 (−0.01, 0.22)<br>*d* = 0.08 (−0.01, 0.17)	MD = 0.07 (−0.04, 0.18)<br>*d* = 0.05 (−0.03, 0.14)	MD = 0.03 (−0.08, 0.15)<br>*d* = 0.03 (−0.06, 0.12)
Distress (NCCN-DT)	2.40 (0.27)	1.22 (0.25)	−1.18††† (0.11)	2.31 (0.26)	1.58 (0.24)	−0.74††† (0.11)	2.70 (0.27)	2.64 (0.24)	−0.06 (0.11)	MD = 1.12 (0.75, 1.48)***<br>*d* = 0.44 (0.30, 0.59)	MD = 0.67 (0.31, 1.03)***<br>*d* = 0.27 (0.12, 0.41)	MD = 0.44 (0.08, 0.81)*<br>*d* = 0.18 (0.03, 0.32)

Note. Estimated marginal means (EMM) with SE, changes and between-arm contrasts from the complete-case model of Table SC.1, averaged equally over the two cycles. Δ = post- minus pre-session change; a negative value denotes improvement. MD, mean difference between arms in within-session change, reported so that a positive value favours the first-named arm; d, the same difference divided by the pooled baseline standard deviation of the full randomised sample, as in main-text Table 3. Confidence intervals are Bonferroni-adjusted 95% intervals for the three pairwise contrasts within each outcome; bounds close to zero are given to three decimals. SE, standard error. Within-group change: †p<.05, ††p<.01, †††p<.001; between-arm, Bonferroni-adjusted: *p<.05, **p<.01, ***p<.001. VR, virtual reality; RV, relaxation video; CG, control group.

Table SC.2. Cycle-specific analysis: Group × Time effects on within-session change

Outcome	Cycle (*n*)	Time main effect			Group × Time				
		F	***df***	***p***	***F***	***df***	***p***	***p(FDR)***	***f***
Pain	1 (315)	37.53	1, 312.0	<.001***	5.05	2, 312.0	.007**	.012*	0.07
	2 (210)	6.87	1, 207.0	.009**	2.47	2, 207.0	.087	.125	0.06
Tiredness	1 (315)	46.34	1, 312.0	<.001***	16.10	2, 312.0	<.001***	<.001***	0.13
	2 (210)	14.80	1, 207.0	<.001***	7.27	2, 207.0	<.001***	.002**	0.11
Nausea	1 (315)	0.62	1, 312.0	.430	2.97	2, 312.0	.053	.075	0.12
	2 (210)	5.05	1, 207.0	.026*	0.59	2, 207.0	.558	.558	0.03
Depression	1 (315)	59.19	1, 312.0	<.001***	14.73	2, 312.0	<.001***	<.001***	0.14
	2 (210)	28.52	1, 207.0	<.001***	11.45	2, 207.0	<.001***	<.001***	0.11
Anxiety	1 (315)	81.30	1, 312.0	<.001***	13.09	2, 312.0	<.001***	<.001***	0.14
	2 (210)	23.82	1, 207.0	<.001***	9.49	2, 207.0	<.001***	<.001***	0.09
Drowsiness	1 (315)	17.74	1, 312.0	<.001***	5.79	2, 312.0	.003**	.007**	0.05
	2 (210)	4.67	1, 207.0	.032*	2.74	2, 207.0	.067	.112	0.03
Lack of appetite	1 (315)	1.99	1, 312.0	.159	0.14	2, 312.0	.871	.871	0.00
	2 (210)	2.75	1, 207.0	.099	0.69	2, 207.0	.502	.558	0.02
Well-being	1 (315)	217.58	1, 312.0	<.001***	55.20	2, 312.0	<.001***	<.001***	0.27
	2 (210)	95.70	1, 207.0	<.001***	23.90	2, 207.0	<.001***	<.001***	0.22
Shortness of breath	1 (315)	2.95	1, 312.0	.087	2.33	2, 312.0	.099	.124	0.02
	2 (210)	2.12	1, 207.0	.146	2.80	2, 207.0	.063	.112	0.04
Numbness	1 (315)	4.19	1, 312.0	.041*	1.82	2, 312.0	.164	.182	0.05
	2 (210)	4.77	1, 207.0	.030*	2.14	2, 207.0	.121	.151	0.06
Distress (NCCN-DT)	1 (315)	122.98	1, 312.0	<.001***	21.99	2, 312.0	<.001***	–	0.18
	2 (210)	64.00	1, 207.0	<.001***	22.69	2, 207.0	<.001***	–	0.17

Note. *F*, *df* and *p* from separate linear mixed models fitted within each cycle, with group, assessment point and their interaction as fixed effects and an unstructured covariance matrix for the two assessments of that cycle. Cycle 1: all 315 randomised participants (VR *n* = 106, RV *n* = 104, CG *n* = 105), with no missing data; cycle 2: the 210 participants who received the second session (VR *n* = 67, RV *n* = 72, CG *n* = 71). Time, within-session change averaged across groups; Group × Time, difference between groups in within-session change in that cycle. *f* is defined as in main-text Table 2, and *p(FDR)* is Benjamini–Hochberg-adjusted across the ten ESAS symptoms within each cycle. Denominator degrees of freedom are Satterthwaite approximations. *p<.05, **p<.01, ***p<.001. NCCN-DT, National Comprehensive Cancer Network Distress Thermometer.

Table SC.2 (continued). Cycle-specific analysis: model-estimated within-session means and changes, and between-group differences in within-session change

		VR			RV			CG			Between-group differences in within-session change (Group × Time)		
**Outcome**	**Cycle (*n*)**	**Pre-EMM (SE)**	**Post-EMM (SE)**	**Change Δ (SE)**	**Pre-EMM (SE)**	**Post-EMM (SE)**	**Change Δ (SE)**	**Pre-EMM (SE)**	**Post-EMM (SE)**	**Change Δ (SE)**	**VR vs CG: MD (95% CI) *d* (95% CI)**	**RV vs CG: MD (95% CI) *d* (95% CI)**	**VR vs RV: MD (95% CI) *d* (95% CI)**
Pain	1 (315)	1.28 (0.22)	0.83 (0.19)	−0.45††† (0.09)	1.16 (0.22)	0.75 (0.19)	−0.41††† (0.09)	1.22 (0.22)	1.13 (0.19)	−0.09 (0.09)	MD = 0.37 (0.06, 0.67)*<br>*d* = 0.16 (0.03, 0.30)	MD = 0.33 (0.02, 0.63)*<br>*d* = 0.15 (0.01, 0.28)	MD = 0.04 (−0.27, 0.34)<br>*d* = 0.02 (−0.12, 0.15)
	2 (210)	0.93 (0.26)	0.63 (0.24)	−0.30†† (0.10)	0.78 (0.25)	0.61 (0.23)	−0.17 (0.10)	1.28 (0.25)	1.30 (0.23)	0.01 (0.10)	MD = 0.31 (−0.03, 0.65)<br>*d* = 0.14 (−0.01, 0.29)	MD = 0.18 (−0.15, 0.52)<br>*d* = 0.08 (−0.07, 0.23)	MD = 0.13 (−0.21, 0.47)<br>*d* = 0.06 (−0.09, 0.21)
Tiredness	1 (315)	2.88 (0.29)	1.90 (0.28)	−0.98††† (0.12)	3.02 (0.29)	2.60 (0.28)	−0.42††† (0.12)	2.75 (0.29)	2.73 (0.28)	−0.02 (0.12)	MD = 0.96 (0.55, 1.37)***<br>*d* = 0.32 (0.18, 0.46)	MD = 0.40 (−0.01, 0.82)<br>*d* = 0.13 (−0.003, 0.27)	MD = 0.56 (0.15, 0.97)**<br>*d* = 0.19 (0.05, 0.32)
	2 (210)	2.85 (0.36)	2.13 (0.34)	−0.72††† (0.15)	3.19 (0.35)	2.88 (0.33)	−0.32† (0.14)	3.03 (0.35)	3.10 (0.33)	0.07 (0.14)	MD = 0.79 (0.29, 1.28)***<br>*d* = 0.26 (0.10, 0.43)	MD = 0.39 (−0.10, 0.88)<br>*d* = 0.13 (−0.03, 0.29)	MD = 0.40 (−0.10, 0.89)<br>*d* = 0.13 (−0.03, 0.30)
Nausea	1 (315)	0.32 (0.10)	0.12 (0.09)	−0.20† (0.09)	0.17 (0.11)	0.28 (0.09)	0.11 (0.09)	0.12 (0.11)	0.10 (0.09)	−0.03 (0.09)	MD = 0.17 (−0.13, 0.47)<br>*d* = 0.16 (−0.12, 0.44)	MD = −0.13 (−0.44, 0.17)<br>*d* = −0.12 (−0.40, 0.16)	MD = 0.30 (0.003, 0.60)*<br>*d* = 0.28 (0.003, 0.56)
	2 (210)	0.21 (0.13)	0.13 (0.12)	−0.07 (0.06)	0.22 (0.12)	0.11 (0.11)	−0.11† (0.05)	0.35 (0.12)	0.32 (0.11)	−0.03 (0.05)	MD = 0.05 (−0.14, 0.24)<br>*d* = 0.04 (−0.13, 0.22)	MD = 0.08 (−0.10, 0.27)<br>*d* = 0.08 (−0.10, 0.25)	MD = −0.04 (−0.22, 0.15)<br>*d* = −0.03 (−0.21, 0.14)
Depression	1 (315)	2.35 (0.26)	1.42 (0.23)	−0.92††† (0.12)	1.98 (0.26)	1.38 (0.23)	−0.60††† (0.12)	2.72 (0.26)	2.69 (0.23)	−0.04 (0.12)	MD = 0.89 (0.49, 1.28)***<br>*d* = 0.33 (0.18, 0.48)	MD = 0.56 (0.16, 0.96)**<br>*d* = 0.21 (0.06, 0.36)	MD = 0.33 (−0.07, 0.73)<br>*d* = 0.12 (−0.03, 0.27)
	2 (210)	2.10 (0.32)	1.57 (0.30)	−0.54††† (0.11)	2.14 (0.31)	1.63 (0.29)	−0.51††† (0.10)	2.24 (0.31)	2.32 (0.30)	0.08 (0.10)	MD = 0.62 (0.26, 0.98)***<br>*d* = 0.23 (0.10, 0.37)	MD = 0.60 (0.25, 0.95)***<br>*d* = 0.23 (0.09, 0.36)	MD = 0.02 (−0.33, 0.38)<br>*d* = 0.01 (−0.13, 0.14)
Anxiety	1 (315)	2.33 (0.27)	1.29 (0.23)	−1.04††† (0.13)	2.42 (0.27)	1.51 (0.24)	−0.91††† (0.13)	2.83 (0.27)	2.69 (0.23)	−0.14 (0.13)	MD = 0.89 (0.44, 1.35)***<br>*d* = 0.33 (0.16, 0.49)	MD = 0.77 (0.31, 1.23)***<br>*d* = 0.28 (0.11, 0.45)	MD = 0.12 (−0.33, 0.58)<br>*d* = 0.05 (−0.12, 0.21)
	2 (210)	2.01 (0.29)	1.46 (0.27)	−0.55††† (0.10)	1.68 (0.28)	1.38 (0.26)	−0.31†† (0.10)	1.92 (0.28)	1.96 (0.26)	0.04 (0.10)	MD = 0.59 (0.26, 0.93)***<br>*d* = 0.22 (0.10, 0.34)	MD = 0.35 (0.02, 0.67)*<br>*d* = 0.13 (0.01, 0.25)	MD = 0.25 (−0.08, 0.58)<br>*d* = 0.09 (−0.03, 0.21)
Drowsiness	1 (315)	2.50 (0.28)	2.12 (0.27)	−0.38††† (0.08)	2.05 (0.28)	1.89 (0.27)	−0.15† (0.08)	2.14 (0.28)	2.12 (0.27)	−0.02 (0.08)	MD = 0.36 (0.10, 0.61)**<br>*d* = 0.13 (0.04, 0.21)	MD = 0.13 (−0.12, 0.39)<br>*d* = 0.05 (−0.04, 0.14)	MD = 0.22 (−0.03, 0.48)<br>*d* = 0.08 (−0.01, 0.17)
	2 (210)	2.70 (0.38)	2.57 (0.38)	−0.13† (0.07)	2.22 (0.37)	2.07 (0.37)	−0.15† (0.06)	3.00 (0.37)	3.04 (0.37)	0.04 (0.06)	MD = 0.18 (−0.05, 0.40)<br>*d* = 0.06 (−0.02, 0.14)	MD = 0.20 (−0.03, 0.42)<br>*d* = 0.07 (−0.01, 0.15)	MD = −0.02 (−0.24, 0.21)<br>*d* = −0.01 (−0.08, 0.07)
Lack of appetite	1 (315)	1.29 (0.27)	1.26 (0.26)	−0.03 (0.04)	1.89 (0.27)	1.88 (0.27)	−0.02 (0.04)	1.98 (0.27)	1.93 (0.27)	−0.05 (0.04)	MD = −0.02 (−0.15, 0.11)<br>*d* = −0.01 (−0.06, 0.04)	MD = −0.03 (−0.16, 0.10)<br>*d* = −0.01 (−0.06, 0.04)	MD = 0.01 (−0.12, 0.14)<br>*d* = 0.00 (−0.05, 0.05)
	2 (210)	1.52 (0.34)	1.52 (0.35)	0.00 (0.10)	2.08 (0.33)	2.24 (0.34)	0.15 (0.10)	1.49 (0.33)	1.62 (0.34)	0.13 (0.10)	MD = 0.13 (−0.21, 0.46)<br>*d* = 0.05 (−0.08, 0.17)	MD = −0.03 (−0.35, 0.30)<br>*d* = −0.01 (−0.13, 0.11)	MD = 0.15 (−0.18, 0.49)<br>*d* = 0.06 (−0.07, 0.18)
Well-being	1 (315)	3.17 (0.21)	1.71 (0.20)	−1.46††† (0.10)	2.99 (0.21)	2.07 (0.20)	−0.92††† (0.10)	3.10 (0.21)	3.05 (0.20)	−0.06 (0.10)	MD = 1.41 (1.08, 1.73)***<br>*d* = 0.66 (0.51, 0.81)	MD = 0.87 (0.54, 1.19)***<br>*d* = 0.41 (0.25, 0.56)	MD = 0.54 (0.21, 0.86)***<br>*d* = 0.25 (0.10, 0.41)
	2 (210)	3.40 (0.26)	2.22 (0.26)	−1.18††† (0.12)	2.89 (0.25)	2.18 (0.25)	−0.71††† (0.11)	3.31 (0.25)	3.25 (0.25)	−0.06 (0.11)	MD = 1.12 (0.73, 1.52)***<br>*d* = 0.53 (0.34, 0.71)	MD = 0.65 (0.26, 1.04)***<br>*d* = 0.31 (0.12, 0.49)	MD = 0.47 (0.08, 0.86)*<br>*d* = 0.22 (0.04, 0.41)
Shortness of breath	1 (315)	0.44 (0.17)	0.36 (0.16)	−0.08†† (0.03)	0.56 (0.17)	0.54 (0.16)	−0.02 (0.03)	0.86 (0.17)	0.87 (0.16)	0.01 (0.03)	MD = 0.09 (−0.01, 0.20)<br>*d* = 0.05 (−0.01, 0.12)	MD = 0.03 (−0.08, 0.14)<br>*d* = 0.02 (−0.05, 0.08)	MD = 0.07 (−0.04, 0.17)<br>*d* = 0.04 (−0.02, 0.10)
	2 (210)	0.33 (0.16)	0.18 (0.15)	−0.15†† (0.05)	0.53 (0.16)	0.53 (0.15)	0.00 (0.05)	0.49 (0.16)	0.51 (0.15)	0.01 (0.05)	MD = 0.16 (−0.02, 0.35)<br>*d* = 0.09 (−0.01, 0.20)	MD = 0.01 (−0.17, 0.19)<br>*d* = 0.01 (−0.10, 0.11)	MD = 0.15 (−0.03, 0.33)<br>*d* = 0.09 (−0.02, 0.19)
Numbness	1 (315)	0.25 (0.12)	0.11 (0.11)	−0.14†† (0.05)	0.31 (0.13)	0.26 (0.11)	−0.05 (0.05)	0.64 (0.13)	0.64 (0.11)	0.00 (0.05)	MD = 0.14 (−0.04, 0.32)<br>*d* = 0.11 (−0.03, 0.25)	MD = 0.05 (−0.13, 0.23)<br>*d* = 0.04 (−0.10, 0.18)	MD = 0.09 (−0.09, 0.28)<br>*d* = 0.07 (−0.07, 0.21)
	2 (210)	0.79 (0.25)	0.61 (0.24)	−0.18†† (0.07)	0.81 (0.24)	0.72 (0.23)	−0.08 (0.06)	0.90 (0.24)	0.92 (0.23)	0.01 (0.07)	MD = 0.19 (−0.03, 0.42)<br>*d* = 0.15 (−0.03, 0.33)	MD = 0.10 (−0.12, 0.32)<br>*d* = 0.08 (−0.10, 0.25)	MD = 0.10 (−0.13, 0.32)<br>*d* = 0.07 (−0.10, 0.25)
Distress (NCCN-DT)	1 (315)	2.22 (0.25)	1.02 (0.22)	−1.20††† (0.12)	2.46 (0.25)	1.50 (0.22)	−0.96††† (0.12)	2.70 (0.25)	2.56 (0.22)	−0.13 (0.12)	MD = 1.06 (0.66, 1.47)***<br>*d* = 0.42 (0.26, 0.58)	MD = 0.83 (0.42, 1.24)***<br>*d* = 0.33 (0.17, 0.49)	MD = 0.24 (−0.17, 0.64)<br>*d* = 0.09 (−0.07, 0.25)
	2 (210)	2.25 (0.32)	1.25 (0.30)	−1.00††† (0.11)	2.31 (0.31)	1.75 (0.29)	−0.56††† (0.11)	2.65 (0.31)	2.69 (0.29)	0.04 (0.11)	MD = 1.04 (0.67, 1.42)***<br>*d* = 0.41 (0.26, 0.56)	MD = 0.60 (0.23, 0.97)***<br>*d* = 0.24 (0.09, 0.38)	MD = 0.44 (0.07, 0.82)*<br>*d* = 0.18 (0.03, 0.32)

Note. Estimated marginal means (EMM) with SE, changes and between-arm contrasts from the cycle-specific models of Table SC.2; arm sizes as in Table SC.2. Δ = post- minus pre-session change; a negative value denotes improvement. MD and *d* are defined as in Table SC.1 (continued), with *d* based on the pooled baseline standard deviation of the full randomised sample. Confidence intervals are Bonferroni-adjusted 95% intervals for the three pairwise contrasts within each outcome; bounds close to zero are given to three decimals. SE, standard error. Within-group change: †*p*<.05, ††*p*<.01, †††*p*<.001; between-arm, Bonferroni-adjusted: **p*<.05, ***p*<.01, ****p*<.001. VR, virtual reality; RV, relaxation video; CG, control group.

Supplementary Section D: Figures

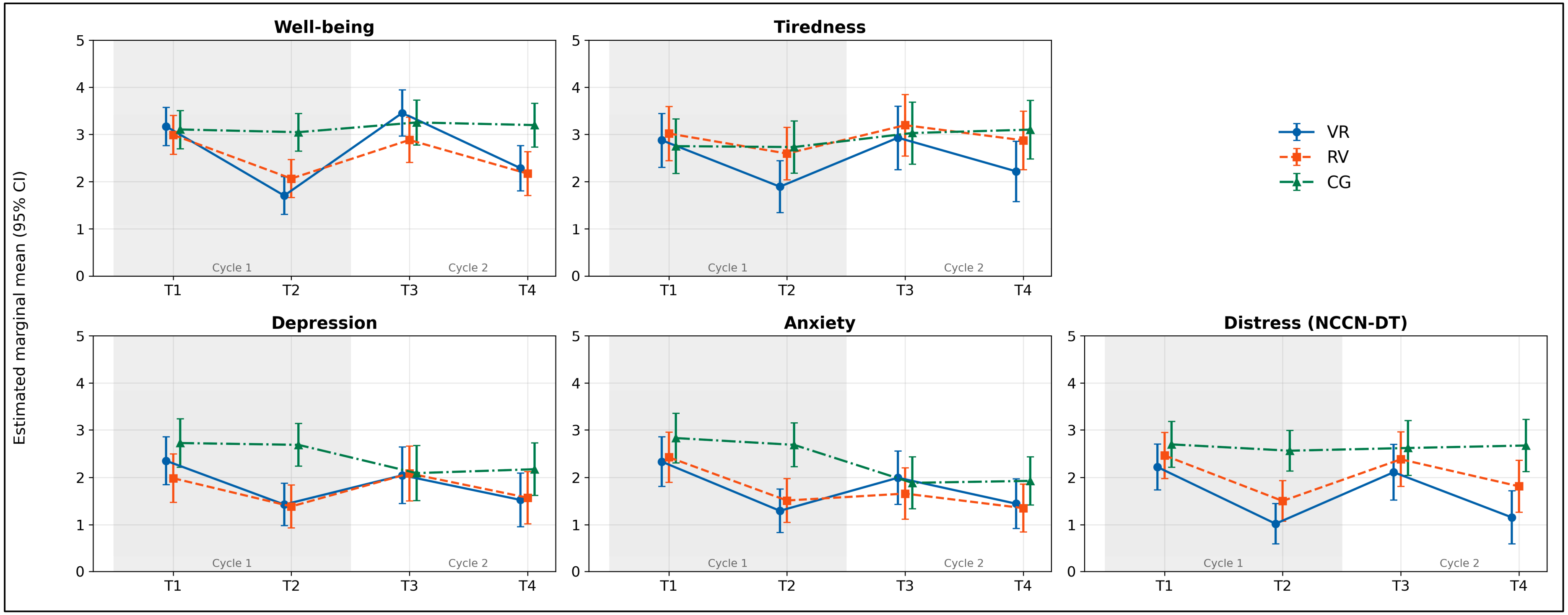

Figure SD.1. Estimated mean trajectories of well-being, tiredness, depression, anxiety and distress across the four assessments.

Note. The four symptoms with the largest responses are shown, together with distress. Points are estimated marginal means with 95% confidence intervals from the linear mixed model of Section 2.7, which uses all available observations without imputation. T1 and T2 are the pre- and post-session assessments of the first chemotherapy cycle, T3 and T4 those of the second; the shaded band marks the first cycle. Lower scores indicate a better state on every panel. CI, confidence interval; NCCN-DT, National Comprehensive Cancer Network Distress Thermometer; VR, virtual reality; RV, relaxation video; CG, control group.

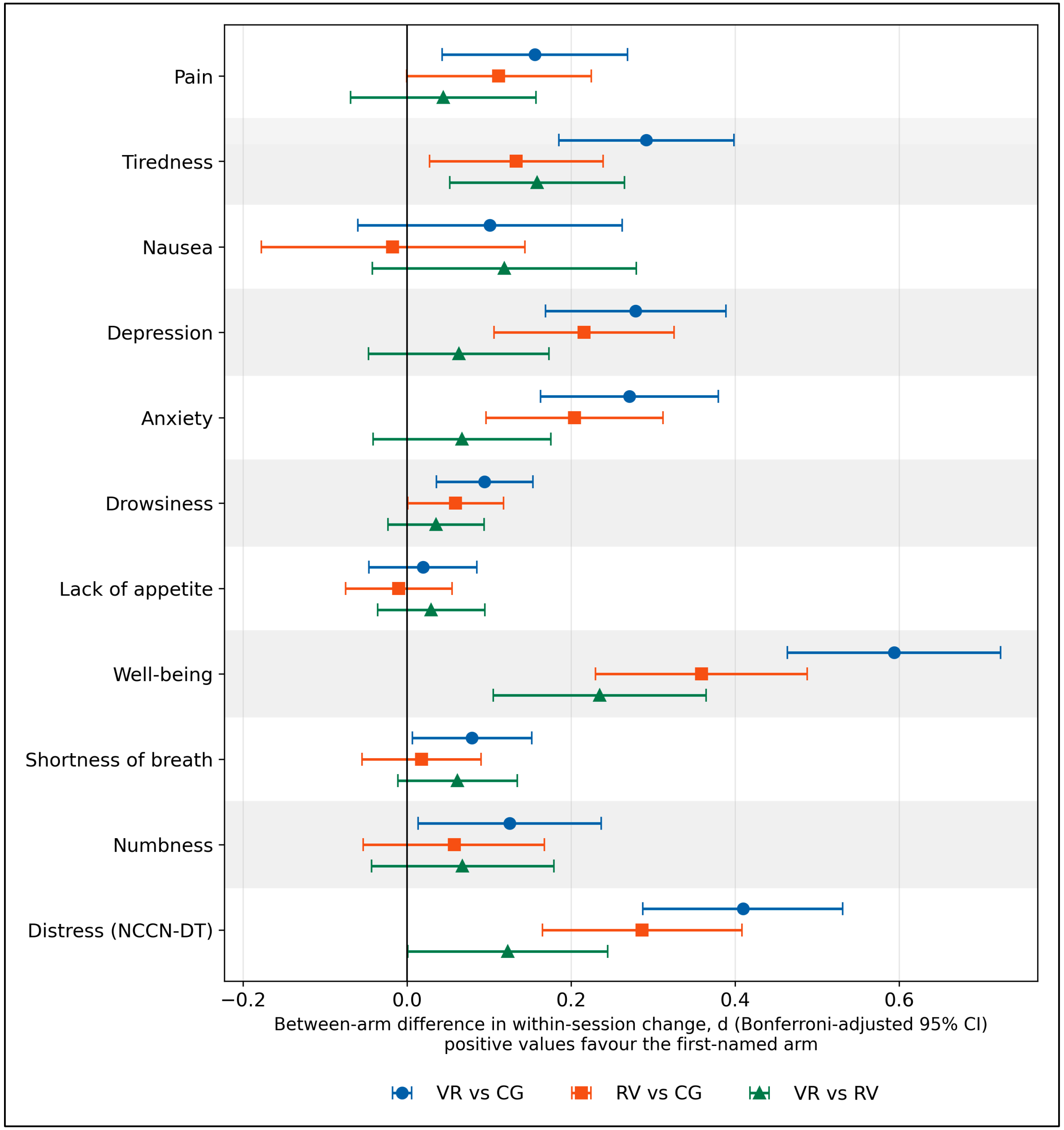

Figure SD.2. Standardised between-arm differences in within-session change for the ESAS symptoms and distress.

Note. Estimates are the pairwise comparisons reported in main-text Table 3, from the linear mixed model described in Section 2.7, each divided by the pooled baseline standard deviation of its own outcome. Intervals are Bonferroni-adjusted 95% intervals, so an interval excluding zero corresponds to an adjusted *p* below .05. Positive values favour the first-named arm. d, standardised difference; CI, confidence interval; ESAS, Edmonton Symptom Assessment Scale; NCCN-DT, National Comprehensive Cancer Network Distress Thermometer; VR, virtual reality; RV, relaxation video; CG, control group.
